# Bone marrow-targetable Green Tea Catechin-Based Micellar Nanocomplex for synergistic therapy of Acute myeloid leukemia

**DOI:** 10.1186/s12951-022-01683-4

**Published:** 2022-11-16

**Authors:** Ki Hyun Bae, Fritz Lai, Jamie Mong, Akiko Niibori-Nambu, Kiat Hwa Chan, Zhisheng Her, Motomi Osato, Min-Han Tan, Qingfeng Chen, Motoichi Kurisawa

**Affiliations:** 1 Institute of Bioengineering and Bioimaging, 31 Biopolis Way, The Nanos, Singapore, 138669 Singapore; 2 Institute of Molecular and Cell Biology, 61 Biopolis Drive, The Proteos, Singapore, 138673 Singapore; 3 Cancer Science Institute of Singapore, National University of Singapore, 14 Medical Drive, Singapore, 117599 Singapore; 4 Division of Science, Yale-NUS College, 16 College Avenue West, Singapore, 138527 Singapore; 5 Present Address: School of Materials Science, Japan Advanced Institute of Science and Technology, 1-1 Asahidai, 923-1292 Nomi, Ishikawa Japan; 6 Present Address: MHT: Lucence Diagnostics, 211 Henderson Rd, #04-02, Singapore, 159552 Singapore

**Keywords:** Bone marrow, Targeting, Sorafenib, Micellar nanocomplex, Leukemia

## Abstract

**Background:**

Currently available anti-leukemia drugs have shown limited success in the treatment of acute myeloid leukemia (AML) due to their poor access to bone marrow niche supporting leukemic cell proliferation.

**Results:**

Herein, we report a bone marrow-targetable green tea catechin-based micellar nanocomplex for synergistic AML therapy. The nanocomplex was found to synergistically amplify the anti-leukemic potency of sorafenib via selective disruption of pro-survival mTOR signaling. In vivo biodistribution study demonstrated about 11-fold greater bone marrow accumulation of the nanocomplex compared to free sorafenib. In AML patient-derived xenograft (AML-PDX) mouse model, administration of the nanocomplex effectively eradicated bone marrow-residing leukemic blasts and improved survival rates without noticeable off-target toxicity.

**Conclusion:**

This study may provide insights into the rational design of nanomedicine platforms enabling bone marrow-targeted delivery of therapeutic agents for the treatment of AML and other bone marrow diseases.

**Graphical Abstract:**

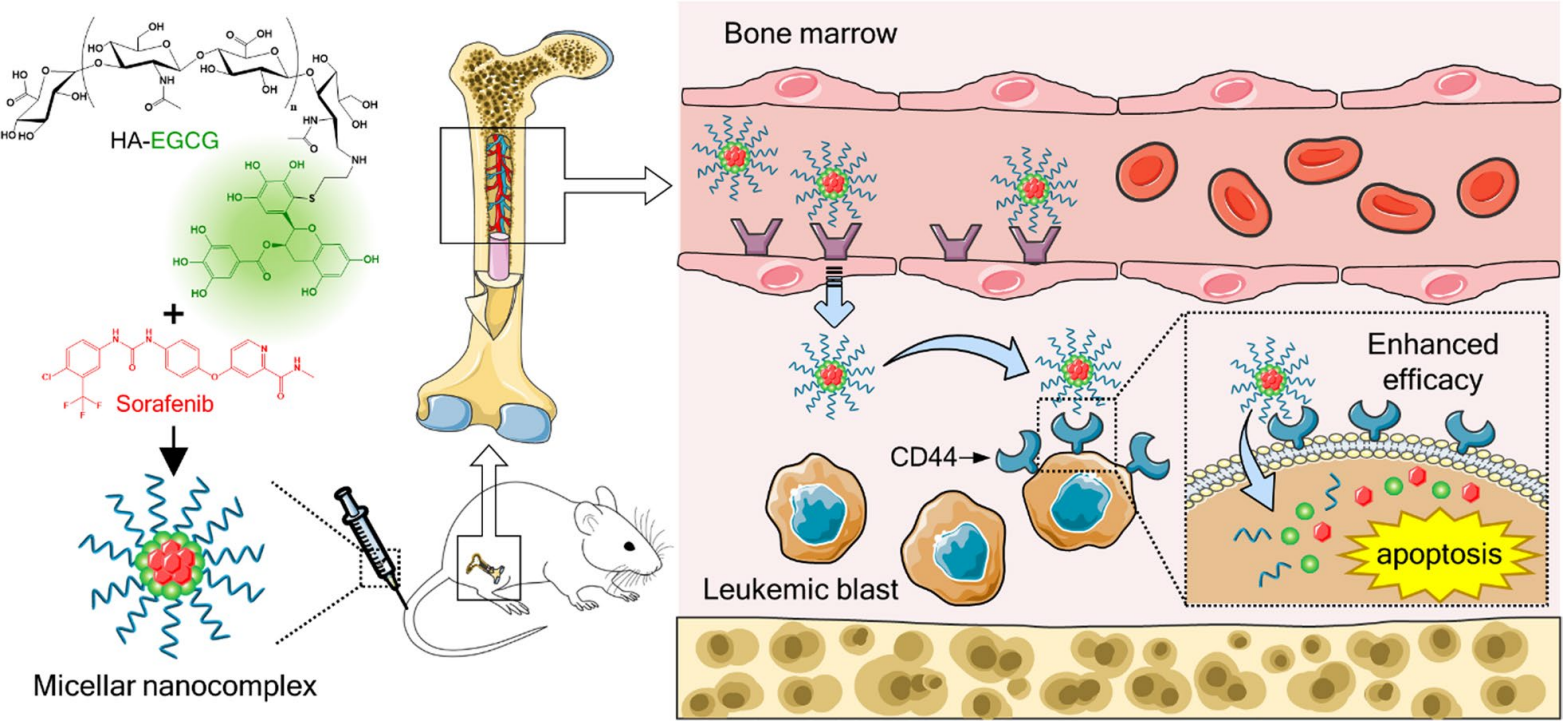

**Supplementary Information:**

The online version contains supplementary material available at 10.1186/s12951-022-01683-4.

## Background

Acute myeloid leukemia (AML) is a highly aggressive form of blood cancer responsible for the majority of leukemia-related deaths [1]. Despite intensive chemotherapy combined with stem cell transplantation, the 5-year survival rate remains poor at 3–8% in patients aged over 60 years and less than 50% in younger patients [2]. Recently, blocking the activity of FMS-like tyrosine kinase 3 (FLT3) has emerged as a promising therapeutic approach because FLT3 mutation is the most frequent genetic alteration conferring poor survival and high risk of relapse [3]. Sorafenib, a multikinase inhibitor with potency against FLT3 mutation, has been clinically used to treat FLT3-positive AML patients in various settings, including front-line, relapsed/refractory, and post-transplant maintenance therapy [4]. However, sorafenib can induce only a transient reduction of leukemic burden because it has limited efficacy on bone marrow-resident AML cells, which are mainly responsible for relapse and treatment failure [5]. Two main reasons have been identified for this challenge: (1) insufficient bone marrow accumulation of sorafenib and (2) elevated drug resistance of AML cells in the bone marrow microenvironment [6, 7]. It is therefore highly desirable to explore the strategy to improve bone marrow targeting and anti-leukemic activity of sorafenib for effective AML therapy.

There has been growing research towards engineering nanoparticles with bone marrow-homing capability. Most of earlier works attempted to improve passive accumulation of nanoparticles by tuning their physiochemical parameters, such as particle size, surface charge and steric stability [8–10]. For example, poly(lactic-*co*-glycolic acid) nanospheres smaller than 150 nm were found to have greater bone marrow uptake than larger ones *via* increased extravasation through the fenestrae of sinusoidal capillaries (85–150 nm in width) [11, 12]. However, due to the lack of cell specificity of passively targeted nanoparticles, recent works have focused on active targeting through functionalization of the particle surface with certain targeting ligands, such as alendronate having bone mineral-binding affinity [13, 14], osteotropic oligopeptide (AspSerSer)_6_ [15], and thioaptamers recognizing E-selectin expressed on bone marrow endothelium [16, 17]. Multiple studies have revealed over-expression of the hyaluronan receptor for endocytosis (HARE) on sinusoidal endothelial cells and its function as an endocytic receptor for hyaluronic acid (HA) *via* strong binding affinity (*K*_d_ = 7 nM) [18–20]. Furthermore, there have been reports on preferential bone marrow localization of intravenously administered HA, highlighting its potential applicability for designing bone marrow-homing nanomedicines [21, 22].

Epigallocatechin-3-*O*-gallate (EGCG) is the primary constituent of green tea catechins possessing antioxidant, anti-inflammatory and cancer-preventive activities [23, 24]. Particularly for AML therapy, EGCG has gained increasing attention because of its ability to differentially induce apoptosis of leukemic blasts rather than normal blood cells [25, 26]. Moreover, EGCG has shown to synergistically augment the efficacy of conventional leukemia drugs including all-*trans*-retinoic acid and arsenic trioxide by interrupting signaling pathways essential for cell survival and proliferation [27, 28]. Such distinctive properties of EGCG provide a unique opportunity for the development of EGCG-based AML therapeutics. We have previously reported that anti-leukemic activity of EGCG can be strengthened by conjugation with HA capable of binding to CD44 overexpressed on AML cells [29]. In this study, we developed bone marrow-targetable micellar nanocomplex (Sora-MNC) via self-assembly of sorafenib and HA-EGCG conjugate for synergistic AML therapy. The nanocomplex showed remarkably enhanced cytotoxicity and anti-clonogenic activity over sorafenib against FLT3-mutated AML cells. When administered in AML patient-derived xenograft (AML-PDX) mouse model, the nanocomplex diminished the leukemia burden in bone marrow and prolonged survival more effectively than free sorafenib formulation, showing promise as a nanomedicine for synergistic AML therapy.

## Materials and methods

### Materials

HA (*M*_w_=20 kDa) was purchased from Lifecore Biomedical (Minnesota, USA). EGCG was purchased from DSM Nutritional Products Ltd (Heerlen, the Netherlands). Sorafenib tosylate was obtained from AbMole BioScience (Houston, USA). Amicon Ultra-15 centrifugal filters were purchased from Merck Millipore Corporation (Darmstadt, Germany). MesenCult-ACF and MethoCult H4434 Classic media were obtained from Stemcell Technologies (Vancouver, Canada). Dextran (*M*_w_=70 kDa) and radioimmunoprecipitation assay (RIPA) buffer was purchased from Sigma-Aldrich (Minnesota, USA). Anti-HARE monoclonal antibody (mAb, MBL International, clone #34 − 2), anti-CD44 mAb (Thermo Fisher, clone Hermes-1) and control rat IgG (Thermo Fisher) were used as received. The following primary antibodies used for Western blotting were rabbit polyclonal unless otherwise stated: phospho-S6 (Ser235/236) and S6 (GeneTex, USA); phospho-STAT5a (Tyr694), STAT5a (C-term), phospho-ERK1/2 (Thr202/Tyr204) and ERK1/2 (Abcepta, USA); β-actin (mouse monoclonal, Proteintech, USA). Secondary antibodies (anti-rabbit/anti-mouse IgG-horseradish peroxidase conjugates) and CellTiter-Glo cell viability assay reagent were purchased from Promega (Madison, USA). RNeasy Mini kit (Qiagen, Germany), QuantiTect Reverse Transcription kit (Qiagen, Germany) and Micro BCA protein assay kit (Thermo Scientific, USA) were used per the manufacturer’s protocol.

### Preparation of Sora-MNC

HA-EGCG conjugate was synthesized according to the previous report [30]. Sorafenib tosylate was dissolved in a 1:1 (v/v) mixture of acetonitrile and methanol. HA-EGCG conjugate solution in deionized water (final concentration: 2–8 mg mL^-1^) was mixed with sorafenib tosylate solution (final concentration: 0.04–0.4 mg mL^-1^) and then incubated for 2 days at 37 °C on an orbital shaker at 50 rpm in a dark place. The mixture was transferred to Amicon Ultra-15 centrifugal filters (*M*_w_ cutoff of 50 kDa). The nanoparticles were retrieved by centrifugation for 5 min at 2,000 × *g* at 25 °C and then purified by repeating dispersion in deionized water and centrifugation three times. The purified nanoparticles were resuspended in 1.5 mL of deionized water and stored at 4 °C until use.

### Characterization of Sora-MNC

The particle size distribution and zeta potential of Sora-MNC were examined using the Nano ZS zetasizer (Malvern Instruments, UK). The stability of Sora-MNC in the presence of serum was evaluated by measuring their Z-average size and zeta potential over 5 days in 10 mM phosphate-buffered saline (PBS, pH 7.4) containing 10% (v/v) fetal bovine serum (FBS). To understand the mechanism of sorafenib-EGCG interactions, the derived count rate and size distribution of Sora-MNC were monitored over 4 days in PBS without or with either 100 mM urea or 0.1% Tween-20. All zetasizer measurements were performed in triplicate at 37 °C. For TEM observation, 10 µL of Sora-MNC sample was added onto a Formvar coated 200-mesh copper grid and allowed to sit for 2 min, after which the excess liquid was blotted off from the opposite face of the grid with filter paper. Then 10 L of 2 aqueous phosphotungstic acid (PTA) was applied on top of the sample on the copper grid. After 2 min, the excess PTA was blotted off. The grid was allowed to dry in air at ambient temperature for 2 h, and then observed under the JEM-2100 F field emission TEM instrument (JEOL, USA) with an accelerating voltage of 200 kV, operating current of 217 µA, and a chamber pressure of 60 µPa. The drug loading efficiency was determined by reversed-phase high-performance liquid chromatography (RP-HPLC), according to the previous report with some modifications [31]. Briefly, 100 µL of each sample was added into 500 µL of acetonitrile/methanol (1:1, v/v) mixture, and then vortexed for 1 h. After centrifugation for 8 min at 10,000×*g* at 4 °C, the supernatant was filtered through the Acrodisc mini-spike GHP filter. The amount of sorafenib in the filtrate was analyzed using the Waters 2695 separation module equipped with the Discovery HS C18 column (5 μm, 25 cm × 4.6 mm). Each sample (20 µL) was eluted with a mobile phase containing acetonitrile and 1% acetic acid (65:35, v/v) at a flow rate of 1 mL min^-1^ at 25 °C. The elution of sorafenib was monitored at 265 nm and analyzed using the Empower 3 software (Waters Corporation, USA). A calibration curve was established using a series of sorafenib tosylate solution (0.4–50 µg mL^-1^). The weight of freeze-dried nanoparticles was also measured to calculate the drug loading content.

### Drug release study

Sora-MNC-1 was transferred to Float-A-Lyzer dialysis tubes with a molecular weight cutoff of 3.5–5 kDa (Spectrum Laboratories, USA). The amount of sorafenib per tube was fixed to 7.5 µg. The dialysis tubes were immersed in total 5 mL of 10 mM PBS (pH 7.4) without or with either 100 mM urea or 0.1% Tween-20, and subsequently incubated at 37 °C on an orbital shaker at 50 rpm with Parafilm sealing to minimize water evaporation. At selected time points, 1 mL of the release fraction was transferred to a quartz cuvette and its UV-visible spectrum was acquired on a Hitachi U-2810 spectrophotometer. After each measurement, the release fraction was returned back to the dialysis tube. The extent of drug release was determined by measuring the absorbance of sorafenib at 265 nm.

### Evaluation of in vitro anti-leukemic activity

The human AML cell lines MOLM-14 and MV-4-11 (ATCC, USA) and patient-derived AML cells [32] were maintained in RPMI 1640 media supplemented with 10% (v/v) FBS and 1% (v/v) penicillin/streptomycin. Human bone marrow stromal cells (Stemcell Technologies, Canada), which are nonleukemic in origin [33], were maintained in MesenCult-ACF media. The cells were seeded on white-walled 96-well plates (10^4^ cells per well) and then incubated in 100 µL of 10% FBS-supplemented media containing Sora-MNC, free sorafenib, HA-EGCG or EGCG at various concentrations. In the case of free sorafenib, a stock solution of sorafenib tosylate was prepared in DMSO and then diluted with RPMI 1640 media to a final DMSO concentration of 1%; this concentration of DMSO had no detectable effect on the leukemic cell growth. After treatment for 3 days, 100 µL of CellTiter-Glo assay reagent was added to each well of the plates. After incubation for 10 min at 25 °C, cellular luminescence was measured using a Tecan Infinite 200 microplate reader (Tecan Group, Switzerland). Results were expressed as percentages of the luminescence signal of analyzed cells relative to untreated controls. To examine the synergism between HA-EGCG and sorafenib, the combination index (CI) values were calculated with the CompuSyn software (ComboSyn Inc., USA) using the median-effect equation [34]. Briefly, cytotoxicity data were plotted using the linearized median effect equation, log(f_a_/f_u_) = m log(D) – m log (D_m_), where f_a_ is the fraction of killed cells, f_u_ is the fraction of survived cells, D is the dose applied, D_m_ is the median effective dose. The resultant plot gave the slope of m and the y-axis intercept of – m log (D_m_). Based on the mutually exclusive model, CI was determined by CI = (D)_1_ / (ED_x_)_1_ + (D)_2_ / (ED_x_)_2_, where (ED_x_)_1_ and (ED_x_)_2_ are the dose of HA-EGCG and sorafenib needed to exert x% effect, respectively. (D)_1_ and (D)_2_ are the dose of HA-EGCG and sorafenib needed to produce the same effect in combination, respectively. The combination was considered synergistic when CI < 1, additive when CI = 1 and antagonistic when CI > 1. For colony forming assay, two to five hundred cells were seeded in 35-mm dishes in Methocult H4434 Classic media containing 1% antibiotic-antimycotic and then treated with either Sora-MNC or free sorafenib at selected concentrations. The number of colonies was counted under an inverted microscope after 14 days of culture.

### Synthesis of DyLight 488-labeled MNC

DyLight 488-labeled HA was prepared according to the previous report [30]. Sorafenib tosylate was dissolved in a 1:1 (v/v) mixture of acetonitrile and methanol. To synthesize DyLight 488-labeled MNC, a mixture of HA-EGCG and DyLight 488-labeled HA (4:1 weight ratio at a final concentration of 8 mg mL^-1^) was added to sorafenib tosylate solution (final concentration: 0.05 mg mL^-1^) and then incubated for 2 days at 37 °C on an orbital shaker at 50 rpm in a dark place. The mixture was transferred to Amicon Ultra-15 centrifugal filters (*M*_w_ cutoff of 50 kDa). The nanoparticles were retrieved by centrifugation for 5 min at 2,000×*g* at 25 °C and then purified by repeating dispersion in deionized water and centrifugation three times. The purified nanoparticles were resuspended in isotonic dextrose solution (5% w/v), sterile-filtered through a 0.45-µm syringe filter and stored at 4 °C until use. The fluorescence spectrum was acquired on a Cary Eclipse fluorescence spectrometer (Agilent) with an excitation wavelength at 400 nm.

### In vitro flow cytometric analysis

To assess the CD44 expression levels, MOLM-14, MV-4-11 and human bone marrow stromal cells (3 × 10^5^ cells) were stained with FITC-tagged anti-human CD44 antibody (clone BJ18, BioLegend) for 1 h at 4 °C. The cells were rinsed three times with ice-cold PBS containing 0.1% (w/v) bovine serum albumin prior to flow cytometry analysis by LSR II flow cytometer (BD Biosciences). To evaluate CD44-targeting ability of Sora-MNC, MOLM-14 and MV-4-11 cells were seeded on 100-mm petri dishes (10^6^ cells per dish) and then pre-treated with 5 mL of serum-free RPMI media containing either HA or dextran at the same concentration (10 mg mL^−1^) for 1 h [35]. Subsequently, 5 mL of serum-free RPMI media containing DyLight 488-labeled MNC (final sorafenib concentration = 12.5 nM) was added to the cells. After 1 h of incubation, the cells were washed with ice-cold PBS three times to remove residual MNC and analyzed by LSR II flow cytometer (BD Biosciences). To evaluate terminal differentiation-inducing effect of Sora-MNC, MOLM-14 and MV-4-11 cells were seeded on 100-mm petri dishes (10^6^ cells per dish) and then incubated in 10 mL of 10% FBS-supplemented RPMI media containing free sorafenib (12.5 nM), Sora-MNC (12.5 nM) or HA-EGCG (134 nM; a dose equivalent to that present in Sora-MNC). All-*trans* retinoic acid (ATRA, 1 µM) was also tested as a positive control. After 3 days of incubation, the cells were harvested and stained with BV510-tagged anti-human CD14 (clone M5E2, BioLegend) and BV421-tagged anti-human CD11b (clone ICRF44, BioLegend) antibodies. CD14^+^CD11b^+^ population was quantified to assess the extent of terminal differentiation of AML cells into monocyte-like cells [36]. Similarly, induction of apoptosis was also quantified by co-staining of FITC-Annexin V (Cat. No. 640,906, BioLegend) and Propidium Iodide (PI) solution (Cat. No. 421,301, BioLegend). Populations that are positive for Annexin V and double-positive for Annexin V and PI represent cells undergoing early and late-stage apoptosis, respectively.

### Western blot analysis

MOLM-14 and MV-4-11 cells were seeded on 100-mm petri dishes (10^6^ cells per dish) and then incubated in 10 mL of 10% FBS-supplemented media containing free sorafenib (12.5 nM), Sora-MNC (12.5 nM), HA-EGCG (134 nM; a dose equivalent to that present in Sora-MNC) or EGCG (134 nM). After treatment for 1 day, the cells were rinsed twice with 10 mL of ice-cold PBS and centrifuged. The resulting cell pellet was resuspended in 200 µL of RIPA buffer supplemented with the Pierce protease/phosphatase inhibitors (Thermo Scientific, USA). After agitation for 30 min at 4 °C, the lysate was centrifuged for 20 min at 10,000×*g* at 4 °C and the resulting supernatant was transferred into Protein LoBind tubes (Eppendorf, Germany). The protein concentration in the supernatant was examined using the Micro BCA protein assay kit. The samples (20 µg proteins per well) were separated by SDS-polyacrylamide gel electrophoresis and then wet-transferred to nitrocellulose membranes. After washing steps, the membranes were probed with antibodies and then developed by the Amersham ECL Prime reagent (GE Healthcare, UK). The chemiluminescence image acquisition and densitometric analysis of p-S6/S6 ratios were performed using the ChemiDoc MP instrument with Image Lab software (Bio-rad, USA).

### RNA sequencing (RNA-seq) analysis

MV-4-11 cells subjected to the same treatment as above were harvested and rinsed twice with 10 mL of ice-cold PBS. Total RNA from three replicates in each group was extracted using the RNeasy Mini kit with DNase incubation for 15 min at 25 °C. RNA was eluted in nuclease-free water and its concentration was determined using the Nanodrop spectrophotometer (Thermo Scientific, USA). The construction of cDNA libraries and sequencing on the NovaSeq 6000 platform were done at NovogeneAIT Genomics (Singapore). Differentially expressed genes (DEGs) with an adjusted *P* value (padj) < 0.05 were identified using the DESeq2 R package (ver. 2_1.6.3). The Venn diagram presenting the number of DEGs in each group was prepared using the function vennDiagram in R. Kyoto Encyclopedia of Genes and Genomes (KEGG) enrichment analysis was performed using the clusterProfiler R package (ver. 2.4.3) to find out which biological pathways are significantly associated with DEGs. The gene expression heat maps were created using R based on the log2 fold change values for each treatment condition relative to the untreated control.

### Quantitative PCR (qPCR) analysis

Total RNA samples were prepared as above and 1 µg of them was converted to cDNA using the QuantiTect Reverse Transcription kit. Then, cDNA was diluted 25 times in nuclease-free water. qPCR was conducted on the ViiA7 real-time PCR system using SYBR Select PCR master mix (Applied Biosystems, USA). Three replicates were in each treatment group and all experiments were conducted three times independently. Each reaction mixture (20 µL) comprised a final concentration of 0.2 µM forward/reverse primer and 5 µL diluted cDNA template. The QuantStudio Real-Time PCR software (Thermo Scientific, USA) was used to determine the threshold for each plate automatically. Cycle threshold (CT) values for each target transcript was normalized to the CT value of the housekeeping gene *ACTB* to obtain dCT values. ddCT was determined against the control group. Relative gene expression is expressed in terms of log2 fold change. The standard deviation (SD) calculation is as follows: $\sqrt{{a}^{2}+{b}^{2}}$ where a = SD of CT(target) and b = SD of CT(*ACTB*).

### Pharmacokinetics and biodistribution analysis

All animal experiments were performed according to the protocols approved by IACUC at the Biological Resource Centre, Singapore. Immunodeficient mice with NOD-*scid Il2rg*^*-/-*^ (NSG) background were purchased from the Jackson Laboratory (Maine, USA). The mice were intravenously injected with isotonic dextrose solution (5% w/v) containing Sora-MNC at a sorafenib dose of 0.4 mg kg^-1^. For comparison, another group of mice received intravenous injections of free sorafenib solution prepared in normal saline/DMSO (95:5, v/v) mixture at an equivalent dose. This concentration of DMSO was reported to cause no appreciable toxicity in mice [37]. At selected time points, blood and organs (kidney, lung, liver, spleen, heart and bone marrow) were collected. For pharmacokinetic analysis, 30 µL of heparinized plasma was added into 270 µL of acetonitrile/methanol (1:1, v/v) mixture and then vortexed for 1 h for extraction of sorafenib [31]. After centrifugation for 8 min at 10,000×*g* at 4 °C, the supernatant was filtered through the Acrodisc mini-spike GHP filter. The amount of sorafenib in the filtrate was analyzed by RP-HPLC as described above. A stock solution of sorafenib tosylate was diluted with blank mouse plasma to prepare a series of calibration standards (0.4–50 µg mL^-1^). The plasma half-life and other pharmacokinetic parameters were calculated with the PKSolver software (China Pharmaceutical University, China). For biodistribution analysis, each organ was mashed through a 70-µm cell strainer, suspended in 2 mL of 1 M Tris-HCl buffer (pH 7.5) and then transferred into 15-mL conical tube. After 1 mL of methanol was added, the solution was subjected to three cycles of extraction with 2 mL of diethyl ether for 20 min and centrifugation for 5 min at 2,000×*g* at 4 °C [38]. The combined organic layers were evaporated under vacuum overnight. The dried residue was reconstituted in 250 µL of methanol with shaking for 20 min and then filtered through the Acrodisc mini-spike GHP filter. The amount of sorafenib in the filtrate was analyzed by RP-HPLC as described above.

### Synthesis of DyLight 800-labeled MNC

DyLight 800-labeled HA was prepared according to the previous report [30]. Sorafenib tosylate was dissolved in a 1:1 (v/v) mixture of acetonitrile and methanol. To synthesize DyLight 800-labeled MNC, a mixture of HA-EGCG and DyLight 800-labeled HA (9:1 weight ratio at a final concentration of 8 mg mL^-1^) was added to sorafenib tosylate solution (final concentration: 0.05 mg mL^-1^) and then incubated for 2 days at 37 °C on an orbital shaker at 50 rpm in a dark place. The mixture was transferred to Amicon Ultra-15 centrifugal filters (*M*_w_ cutoff of 50 kDa). The nanoparticles were retrieved by centrifugation for 5 min at 2,000×*g* at 25 °C and then purified by repeating dispersion in deionized water and centrifugation three times. The purified nanoparticles were resuspended in isotonic dextrose solution (5% w/v), sterile-filtered through a 0.45-µm syringe filter and stored at 4 °C until use. The fluorescence spectrum was acquired on a Cary Eclipse fluorescence spectrometer (Agilent) with an excitation wavelength at 750 nm.

### In vivo fluorescence imaging analysis

NSG mice were injected intravenously with isotonic dextrose solution (5% w/v) containing DyLight 800-labeled MNC at a sorafenib dose of 0.4 mg kg^-1^. At selected time points, near-infrared fluorescence images of the mice were acquired using the IVIS Spectrum in vivo imaging system (PerkinElmer, USA). For antibody blocking study, NSG mice received intraperitoneal injection of anti-HARE mAb, anti-CD44 mAb or control IgG at a dose of 3 mg/kg, followed 2 days later by intravenous administration of DyLight 800-labeled MNC (sorafenib dose = 0.4 mg kg^-1^), according to a previously reported method [39]. At 4 h post-injection, organs (kidney, lung, liver, spleen, heart) and femur and tibia bones were collected for ex vivo imaging. The total radiant efficiency in each region of interest was quantified using the Living Image software (PerkinElmer, USA).

### In vivo therapeutic efficacy study

A pre-clinical patient-derived liquid xenograft mouse model was established based on the previous report [32]. Briefly, NSG mice were sub-lethally irradiated at 1 Gy and engrafted with CD34^+^, CD3-depleted bone marrow mononuclear cells from AML patient. AML chimerism was measured by flow cytometry analysis of the percentage of human CD45^+^ (hCD45^+^) cells over total CD45 (human + mouse) population in peripheral blood. When the proportion of hCD45^+^ cells in peripheral blood reached around 10–15%, the mice were randomly divided into 3 groups. The first group received intravenous injections of isotonic dextrose solution containing Sora-MNC at a sorafenib dose of 0.4 mg kg^-1^ twice weekly for 4 weeks. The second group received intravenous injections of free sorafenib solution prepared in normal saline/DMSO (95:5, v/v) mixture at an equivalent dose. The last group did not receive any treatment as a control. At selected time points, mice were bled submandibularly to examine the proportion of hCD45^+^ cells in peripheral blood. The survival time and body weight were monitored throughout the experiment. At the end of the study, the mice were sacrificed to collect peripheral blood, spleen and bone marrow samples.

### Flow cytometry analysis of peripheral blood, spleen and bone marrow

Peripheral blood was collected in EDTA-containing tubes and red blood cells (RBC) were lyzed using RBC lysis buffer (Life Technologies, USA). Mouse spleens were mashed through a 70-µm cell strainer followed by RBC lysis. Bone marrow was collected by flushing cellular contents out of the femur and tibia from hind legs of each mouse. The bone marrow cells were similarly filtered through a 70 μm cell strainer followed by RBC lysis. The collected cells were incubated with anti-hCD45 (HI30; Biolegend) and anti-mouse CD45.1 (A20; BD Biosciences) monoclonal antibodies for 30 min at room temperature prior to flow cytometry data acquisition using LSR II flow cytometer (BD Biosciences). Data was analyzed using FlowJo software (version 10; Tree Star Inc).

### Blood chemistry and histological examination

For blood chemistry analysis, peripheral blood was collected in heparinized tubes and then examined with a VetScan VS2 blood chemistry analyzer (Abaxis, USA). For histological analysis, forelimbs of each mouse were collected, fixed in 10% formalin, decalcified with osteosoft, and processed prior to embedment into paraffin blocks. Each block was sectioned at ~ 5 μm thickness and subjected to Hematoxylin & Eosin (H&E) staining and immunohistochemistry (IHC) with hCD45 antibody (Abcam, ab781). The primary antibody was detected using Mouse Polymer IHC Kit (Abcam) according to the manufacturer’s instructions. Histopathological images were acquired using Axio Scan Z1 slide scanner (Zeiss) and analyzed using Zen 2 (blue edition; Zeiss) software.

### Hemolysis assay

The peripheral blood of healthy NSG mice was collected as described above and then diluted with 10 mM PBS (pH 7.4) to obtain a density of 2 × 10^8^ RBC/mL. The diluted RBC (0.5 mL) were mixed with 0.5 mL of Sora-MNC-1 dispersion at various sorafenib doses. RBC dispersed in PBS was used as a negative control, while RBC dispersed in 1% Triton X-100 was used as a positive control. The mixtures were incubated for 1 h at 37 °C and then subjected to centrifugation for 5 min at 1,000×*g* at 4 °C. After 100 µL of the supernatant was transferred to each well of 96-well plates, absorbance at 576 nm (an indicator of hemoglobin leakage from RBC) was measured using a Tecan Infinite 200 microplate reader (Tecan Group, Switzerland).

### Statistical analysis

All data are presented as mean ± standard deviation (SD). Statistical analysis was conducted using the OriginPro 9 software (one-way ANOVA with Tukey’s post hoc multiple comparison test). The Kaplan-Meier survival curves were analyzed by a log-rank test. Significance was determined at *P* values smaller than 0.05.

## Results and discussion

### Formulation and characterization of Sora-MNC

**Fig. 1:**
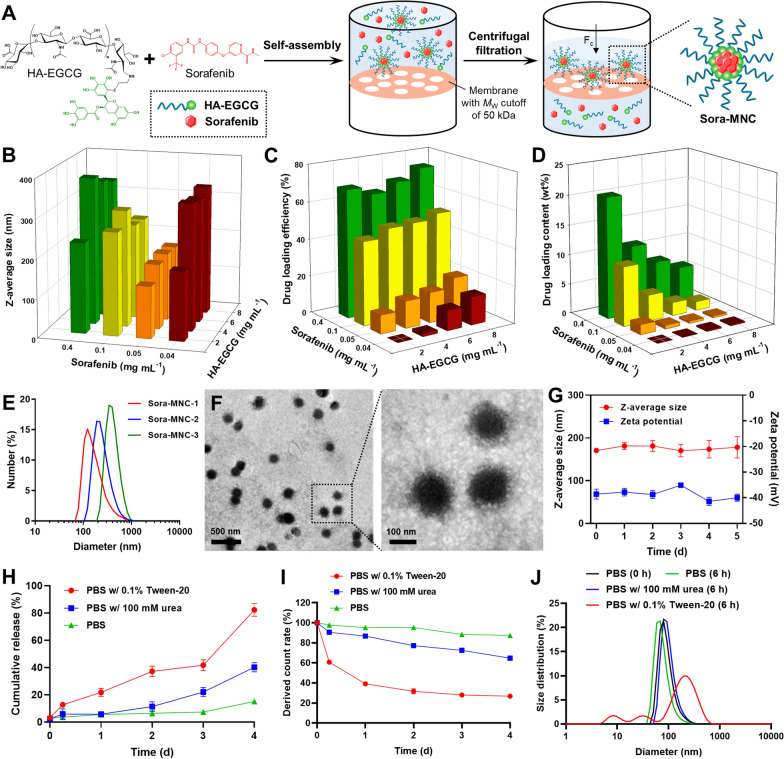
Formulation and characterization of Sora-MNC. **A** Schematic diagram illustrating the self-assembly of Sora-MNC and centrifugal filtration process for purification. **B** Z-average size, **C** drug loading efficiency and **D** drug loading content of Sora-MNC prepared from HA-EGCG and sorafenib at varying final concentrations. Results are reported as mean values (*n* = 3). **E** Particle size distribution and **F** TEM images of Sora-MNC-1. **G** Stability of Sora-MNC-1 in 10% (v/v) fetal bovine serum over 5 days. **H** Cumulative sorafenib release and **I** derived count rate of Sora-MNC-1 over 4 d incubation in 10 mM PBS (pH 7.4) without or with either 100 mM urea or 0.1% Tween-20. Mean ± SD (*n* = 3). **J** Particle size distribution of Sora-MNC-1 measured after 6 h incubation in 10 mM PBS (pH 7.4) without or with either 100 mM urea or 0.1% Tween-20

Figure 1A illustrates the formation of micellar nanocomplex via self-assembly of sorafenib and HA-EGCG conjugate for effective AML therapy. HA-EGCG conjugate was synthesized by conjugating thiol end-modified HA with a single EGCG molecule through nucleophilic addition mechanism [30]. Simple mixing of sorafenib and HA-EGCG solution led to spontaneous self-assembly of sorafenib/HA-EGCG micellar nanocomplex, termed Sora-MNC, via multiple noncovalent interactions between sorafenib and EGCG moieties. The subsequent centrifugal filtration allows rapid drainage of water to concentrate Sora-MNC, which are much larger than the *M*_w_ cutoff of 50 kDa, while ensuring efficient removal of uncomplexed sorafenib (464.8 Da) and HA-EGCG conjugate (~ 20.5 kDa) from the mixture [30]. The inner core of Sora-MNC containing closely packed EGCG moieties can serve as a reservoir for encapsulation of sorafenib due to their capability to form various intermolecular interactions, such as hydrogen bonding, π-π stacking and hydrophobic interactions [40–42]. On the other hand, the outer HA shell layer of Sora-MNC is designed to facilitate its preferential bone marrow accumulation *via* recognition of HARE on the sinusoidal endothelial cells [18–20]. CD44-binding ability of HA is another advantage that enables selective delivery of sorafenib and EGCG into CD44-overexpressing AML cells to achieve synergistic anti-leukemic action.

We investigate the formation of Sora-MNC under various concentrations of sorafenib and HA-EGCG by examining their particle size and drug loading capacity. Dynamic light scattering (DLS) analysis showed that the concentration of sorafenib had a significant influence on the self-assembly of Sora-MNC (Fig. 1B). For instance, the Z-average size of Sora-MNC markedly decreased from ~ 330 nm to ~ 120–170 nm when sorafenib concentration was raised from 0.04 to 0.05 mg mL^-1^, suggesting the formation of more compact nanocomplex via enhanced sorafenib-EGCG interactions at the higher sorafenib concentration. However, a further increase in sorafenib concentration caused substantial increment in particle sizes beyond 200 nm, probably due to excessive binding of sorafenib to EGCG moieties. Reversed-phase HPLC was employed to measure the drug loading efficiency (Fig. 1C) and loading content (Fig. 1D) of Sora-MNC. Generally, raising HA-EGCG concentration gradually escalated the drug loading efficiency with a concomitant decline in the drug content. The improved sorafenib encapsulation was likely attributed to the ability of EGCG moieties to form hydrophobic and other noncovalent interactions [40–42]. Multiple studies have documented that both particle size and surface charge are key factors in determining the cellular uptake of nanomedicine [43–45]. For instance, polymeric nanoparticles averaging ~ 100 nm have been shown to allow efficient endocytosis, while bigger particles are less efficient in cellular uptake [44]. Additionally, positively charged nanoparticles usually exhibit faster internalization than neutral and negatively charged ones [45]. In this study, based on the above screening results, three nanocomplex formulations termed Sora-MNC-1 to -3 (Fig. 1E) were chosen to investigate the effect of particle size on the in vitro anti-leukemic activity. Sora-MNC-1, -2 and -3 had different particle sizes of ca. 122, 190 and 342 nm, respectively, while their zeta potential values were not significantly different (Additional file 1: Table S1). TEM visualized the spherical core-shell structure of Sora-MNC appearing as a darker, electron-dense core surrounded by a lighter shell layer (Fig. 1F and Additional file 1: Fig. S1), verifying the presence of external HA chains around closely packed EGCG moieties. This finding was corroborated by the highly negative zeta potential (−35 to −42 mV) of Sora-MNC, which supports the existence of anionic HA molecules on the particle surface (Additional file 1: Fig. S2). As shown in Fig. 1G, Sora-MNC-1 did not show noticeable change in Z-average size and zeta potential in 10% (v/v) fetal bovine serum over 5 days, indicative of its high stability under the physiological environment. In contrast, HA-EGCG micelles self-assembled in the absence of sorafenib were unstable in 10% (v/v) fetal bovine serum (Additional file 1: Fig. S3), implying that sorafenib-EGCG interactions contributed to the observed enhanced stability of Sora-MNC.

To investigate the mechanism of sorafenib-EGCG interactions, sorafenib release study was conducted in PBS containing Tween-20 (surfactant) or urea (hydrogen bond-breaker). As depicted in Fig. 1H, Sora-MNC exhibited a more pronounced release of sorafenib in Tween-20-supplemented PBS when compared to PBS alone, indicating that the sorafenib-EGCG interactions were effectively disrupted by Tween-20 probably via hydrophobic competition [40]. A moderate level of sorafenib release was observed in urea-supplemented PBS, suggesting that the self-assembly of Sora-MNC was governed, at least partly, by hydrogen bonding between sorafenib and EGCG. This finding was further confirmed by DLS analysis, in which Tween-20 caused a faster decrease in derived count rates (absolute light scattering intensities) than urea, reflecting accelerated disintegration of Sora-MNC in the presence of Tween-20 (Fig. 1I). Moreover, the size distribution of Sora-MNC (peaked at 93 nm) was only slightly shifted by incubation with urea for 6 h, whereas the treatment of Tween-20 led to the formation of heterogeneous particles (peaked at 32 and 197 nm) during the same period of time (Fig. 1J). The small peak of 8.3 nm observed in Tween-20-supplemented PBS was likely originated from residual Tween-20 micelles [46]. Collectively, it was conceivable that hydrophobic interactions between sorafenib and EGCG, rather than hydrogen bonding, predominantly contributed to the effective incorporation of sorafenib within the MNC structure.

### Therapeutic effects of Sora-MNC in vitro

**Fig. 2:**
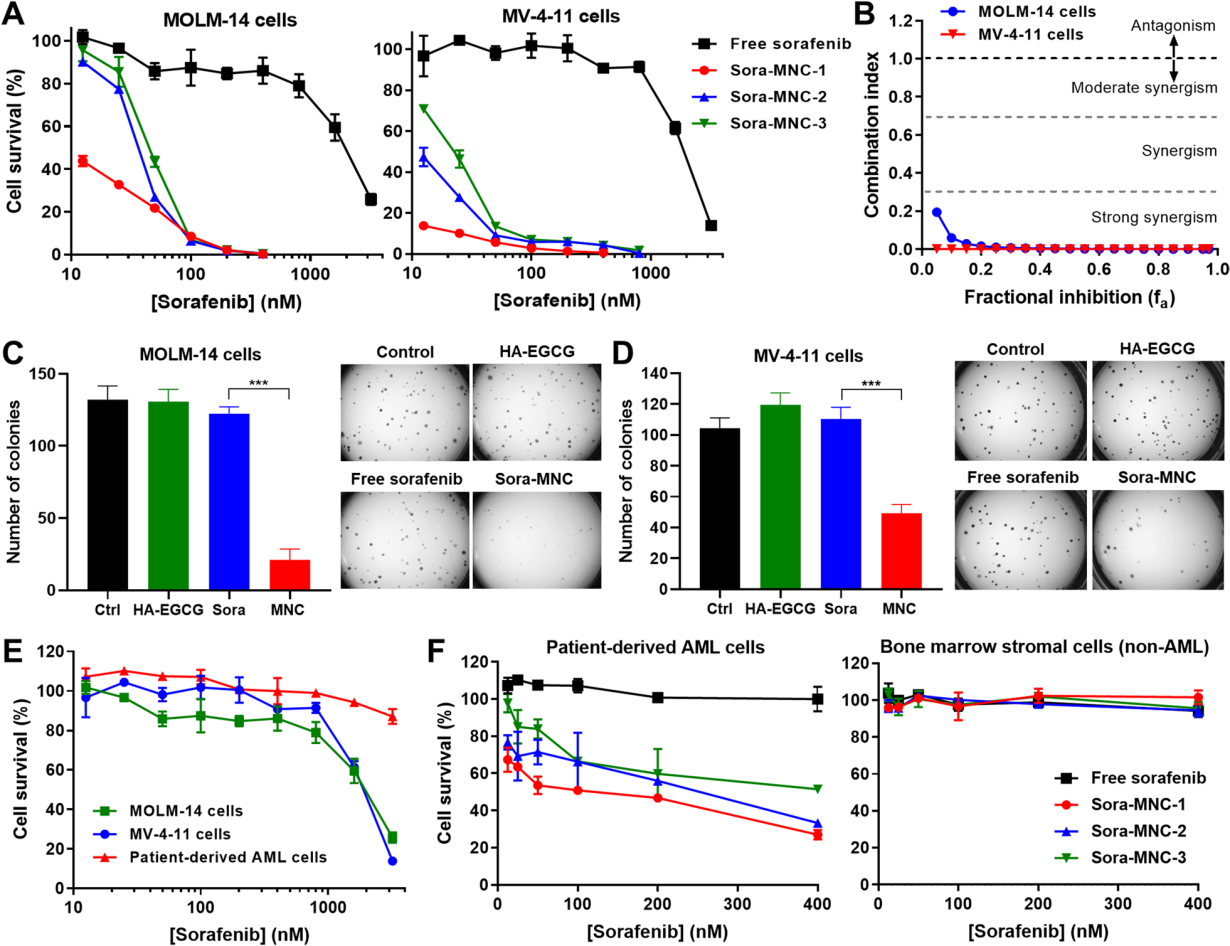
In vitro anti-leukemic effect. **A** Cytotoxicity of Sora-MNC and free sorafenib on MOLM-14 and MV-4-11 cells. Mean ± SD (*n* = 4). **B** Combination index plot. Colony formation of **C** MOLM-14 and **D** MV-4-11 cells treated with Sora-MNC, free sorafenib or HA-EGCG at equivalent doses. The dose of sorafenib for MOLM-14 and MV-4-11 cells was 50 and 200 nM, respectively. The right panels show the colonies formed after 14 days of culture. Mean ± SD (*n* = 3); ^***^P < 0.001. **E** Viability of MOLM-14, MV-4-11 and patient-derived AML cells treated with free sorafenib at varying concentrations. **F** Viability of patient-derived AML cells and bone marrow stromal cells treated with Sora-MNC or free sorafenib at varying concentrations. Mean ± SD (*n* = 4)

In vitro anti-leukemic efficacy of Sora-MNC was evaluated on two different FLT3-mutated AML cell lines: MOLM-14 and MV-4-11 cells (Fig. 2 A). Notably, all Sora-MNC formulations were much more efficacious in killing AML cells than free sorafenib at equivalent doses. For example, Sora-MNC eliminated over 99% of MOLM-14 cells at 200 nM, whereas the same dose of free sorafenib caused only a modest reduction (~ 14%) of the cell viability. Interestingly, the order of effectiveness (Sora-MNC-1 > Sora-MNC-2 > Sora-MNC-3) was inversely correlated with the order of particle size (Sora-MNC-1: 122 nm < Sora-MNC-2: 190 nm < Sora-MNC-3: 342 nm). The strongest anti-leukemic activity of Sora-MNC-1 was probably ascribed to its smallest particle size, which is favorable for intracellular transport [44, 45]. Cytotoxicity of Sora-MNC-1 was far greater than that of HA-EGCG, reflecting the synergistic action between sorafenib and HA-EGCG (Additional file 1: Fig. S4). To examine the level of synergy, the combination index (CI) was calculated using the median-effect plot analysis [34] (Additional file 1: Fig. S5). Strong synergy (CI < 0.3) was observed for both MOLM-14 and MV-4-11 cells, implying that Sora-MNC-1 exerted synergistic anti-leukemic activity via co-delivery of sorafenib and HA-EGCG (Fig. 2B). This strong synergy would be beneficial for AML therapy because it helps achieve desired therapeutic efficacy at lower doses of sorafenib, thus preventing dose-related adverse drug reactions. Colony formation assay was conducted to assess the effect of Sora-MNC-1 on clonogenic progenitor cells, which are capable of initiating leukemic hematopoiesis [47]. Sora-MNC-1 markedly abrogated the colony formation in both MOLM-14 and MV-4-11 cells, while no inhibition was detected for sorafenib and HA-EGCG, demonstrating superior anti-clonogenic activity of Sora-MNC-1 (Fig. 2 C and 2D). To further validate the potential of Sora-MNC, we challenged to treat patient-derived AML cells [32], which showed higher resistance to sorafenib than MOLM-14 and MV-4-11 cells (Fig. 2E). Impressively, Sora-MNC eradicated patient-derived AML cells more substantially than free sorafenib without affecting nonleukemic bone marrow stromal cells (Fig. 2F). Negligible hemolysis was observed in the red blood cells treated with Sora-MNC over a broad dose range (12.5–400 nM), indicating its good hemocompatibility (Additional file 1: Fig. S6). These results suggest that Sora-MNC has the ability to kill AML cell lines and patient leukemic blasts, while not being harmful to healthy normal cells. Sora-MNC-1 having the strongest potency was selected for the rest of studies.

### In vitro flow cytometric analysis

**Fig. 3:**
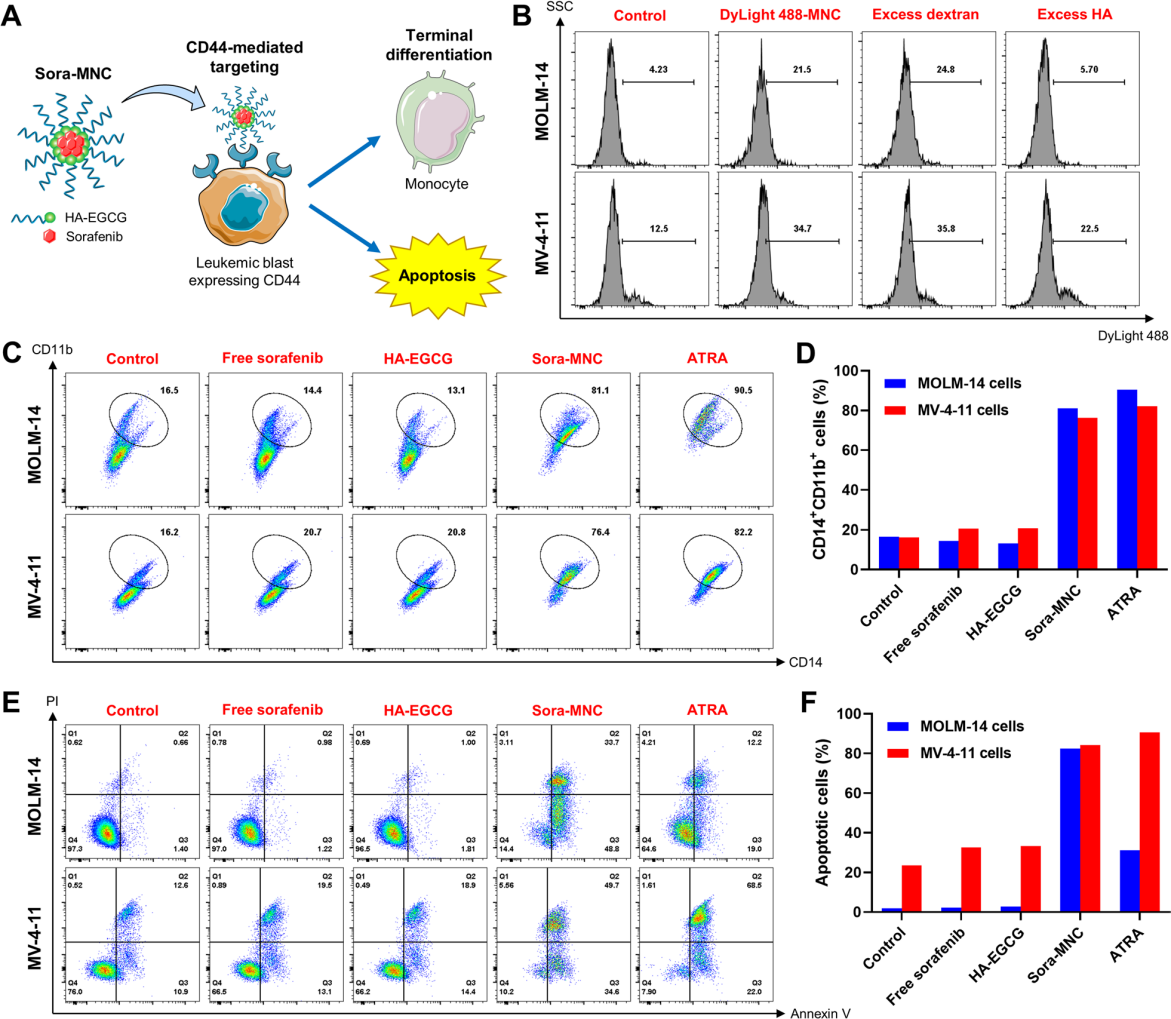
Flow cytometric analysis. **A** Scheme for CD44-mediated targeting of Sora-MNC and induction of terminal differentiation and apoptosis. **B **Cellular binding of DyLight488-labeled MNC on MOLM-14 and MV-4-11 cells after 1 h incubation without or with excess dextran or HA. **C** Representative scatter plots and **D** quantification of CD14^+^CD11b^+^ population in MOLM-14 and MV-4-11 cells treated with free sorafenib, HA-EGCG, Sora-MNC or ATRA. **E** Representative scatter plots of FITC-Annexin V/PI staining and **F** quantification of apoptotic population in MOLM-14 and MV-4-11 cells treated with free sorafenib, HA-EGCG, Sora-MNC or ATRA.

In recent years, EGCG has received growing attention for its potential use in the treatment of AML via a two-pronged approach: induction of terminal differentiation and concurrent apoptosis of myeloid blast cells [48–50]. However, high doses (25–100 µM) of EGCG are typically required to produce the desired anti-leukemic effect, possibly due to the lack of leukemic cell-targeting ability [48]. We hypothesized that Sora-MNC would selectively recognize the cell-surface CD44 receptor expressed on AML cells via HA-CD44 interactions, thus enabling EGCG payload to internalize the cells at lower doses and enhancing its therapeutic efficacy (Fig. 3A). To verify the hypothesis, DyLight 488-labeled MNC emitting green fluorescence was synthesized and treated to MOLM-14 and MV-4-11 cells, which had high levels of CD44 expression (Additional file 1: Fig. S7). DyLight 488-labeled MNC had similar Z-average size and zeta potential as those of unlabeled ones, indicating that the fluorescent labeling had little influence on the particle structures (Additional file 1: Fig. S8). Flow cytometric analysis revealed that cellular fluorescence of both MOLM and MV-4-11 cells increased after 1 h incubation with DyLight 488-labeled MNC (Fig. 3B). The pretreatment of excess HA as a CD44 blocker markedly diminished the cellular entry of DyLight 488-labeled MNC, whereas negligible change was observed upon addition of excess dextran (non-CD44 blocking polymer), demonstrating CD44-targeting ability of MNC.

Next, we assessed the co-expression of CD14 (a monocyte marker) and CD11b (a pan-myeloid marker) of AML cells to investigate the ability of Sora-MNC to reverse their differentiation blockage. All-*trans* retinoic acid (ATRA), which has been clinically used for the treatment of acute promyelocytic leukemia (a subtype of AML), was given at its pharmacological dose (1 µM) as a positive control [51]. As presented in Fig. 3C, Sora-MNC treatment led to the emergence of CD14^+^CD11b^+^ population in both MOLM and MV-4-11 cells, indicative of their terminal differentiation into monocyte-like cells. In contrast, the same dose of free sorafenib (12.5 nM) or HA-EGCG (134 nM) brought only a marginal change to CD14^+^CD11b^+^ population. It was encouraging to note that the differentiation-inducing ability of Sora-MNC was comparable to those of the clinically approved drug ATRA (Fig. 3D). The induction of cellular apoptosis was also investigated by FITC-Annexin V/PI staining. Sora-MNC caused a dramatic rise in early apoptotic (Annexin V^+^, PI^-^) and late apoptotic (Annexin V^+^, PI^+^) population in both MOLM and MV-4-11 cells (Fig. 3E). More than 80% of the cells underwent early and late apoptosis upon treatment with Sora-MNC, confirming that apoptosis was the main mechanism of MNC-induced cell death (Fig. 3F). Of note, Sora-MNC was much more effective in triggering apoptosis of AML cells than free sorafenib and HA-EGCG at an equivalent dose. Taken together, the above results revealed that Sora-MNC exerted synergistic anti-leukemic effects through induction of terminal differentiation and apoptosis in AML cells.

### Mechanism of anti-leukemic activity of Sora-MNC

**Fig. 4:**
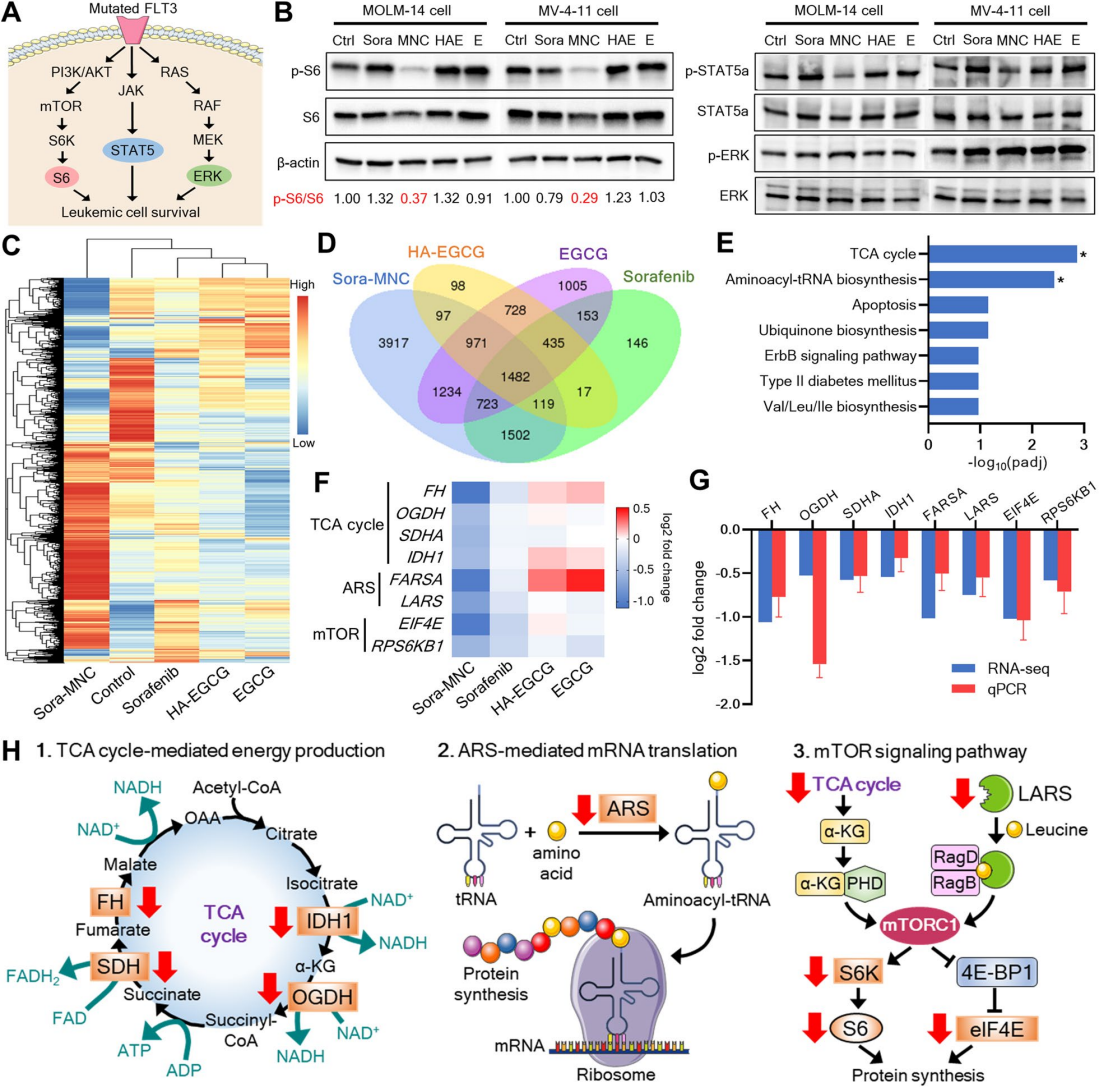
Effect of Sora-MNC on FLT3 signaling and gene expression profile. **A** Scheme of three major downstream signaling pathways of FLT3. **B** Western blot of MOLM-14 and MV-4-11 cells subjected to different treatments (Sora: free sorafenib, MNC: Sora-MNC, HAE: HA-EGCG, E: EGCG, Ctrl: untreated control). The numbers represent p-S6/S6 ratios. **C** Hierarchical clustering heatmap of differentially expressed genes (DEGs). Gradient scale represents upregulation (red) and downregulation (blue). **D** Venn diagram showing the number of DEGs in each experimental group and the overlaps between groups. **E** The top seven pathways identified by KEGG enrichment analysis. Asterisks indicate significantly enriched pathways with an adjusted P value (padj) < 0.05. **F** Heatmap of representative DEGs involved in the TCA cycle, aminoacyl-tRNA synthetase (ARS) and mTOR signaling. **G** qPCR validation of RNA-seq data. Mean ± SD (*n* = 3). **H** Scheme of the mechanism of anti-leukemic action of Sora-MNC. Red arrows highlight downregulation of gene expression in response to Sora-MNC treatment

We reasoned that the observed anti-leukemic activity of Sora-MNC might be correlated with the alteration of FLT3 signaling pathways because mutated FLT3 has been recognized as a main therapeutic target of sorafenib [4]. To address this question, we performed Western blot analysis of the three major downstream effectors of FLT3 signaling pathway: S6, STAT5 and ERK (Fig. 4A), which are known to drive leukemic cell growth [52]. Interestingly, Sora-MNC abolished the phosphorylation of S6 more preferentially than STAT5a and ERK in both MOLM-14 and MV-4-11 cells, as evident from a remarkable decrease in p-S6/S6 ratio (Fig. 4B). On the other hand, the same dose of free sorafenib, HA-EGCG and EGCG had little influence on the phosphorylation levels of S6, STAT5 and ERK. These results suggest that the anti-leukemic activity of Sora-MNC might be closely related with selective inhibition of pro-survival mTOR signaling pathway and its downstream molecules.

We next conducted RNA sequencing (RNA-seq) analysis to better understand the mechanism of action of Sora-MNC. Of note, the gene expression profile of MNC-treated cells was distinctly different from those treated with free sorafenib, HA-EGCG or EGCG (Fig. 4C). From enrichment analysis of total 3917 genes exclusively regulated by Sora-MNC (Fig. 4D), we discovered that TCA cycle and aminoacyl-tRNA biosynthesis pathways were significantly associated with the anti-leukemic action of Sora-MNC (Fig. 4E). Indeed, Sora-MNC caused substantial downregulation of multiple genes involved in the TCA cycle, aminoacyl-tRNA synthetase (ARS) and mTOR signaling, while such effect was not seen for free sorafenib, HA-EGCG and EGCG (Fig. 4F, Additional file 1: Figs. S9 and S10). This finding was further supported by qPCR, which detected the same trend of gene expression as those determined in RNA-seq analysis (Fig. 4G and Additional file 1: Table S2). Overall, the mechanism of action of Sora-MNC appears to involve the disruption of three biological pathways: (1) TCA cycle-mediated energy production, (2) ARS-mediated mRNA translation and (3) mTOR signaling pathway (Fig. 4H). First, Sora-MNC downregulated the genes encoding TCA cycle enzymes essential for production of cellular energy sources (ATP, NADH and FADH_2_) [53]. Second, Sora-MNC abrogated the expression of ARS, such as leucyl-tRNA synthetase (LARS), which catalyzes aminoacyl-tRNA formation for protein synthesis [54]. Lastly, Sora-MNC repressed mTOR signaling via cooperative modulation of TCA cycle and LARS. For example, blockade of TCA cycle is known to diminish the production of α-ketoglutarate (α-KG) capable of activating mTOR complex 1 (mTORC1) [55]. Moreover, mTORC1 activity can be suppressed by interfering with LARS, which governs leucine-dependent mTORC1 activation [56]. This was well corroborated by decreased expression of downstream genes of mTOR pathway (*S6K*, *S6* and *eIF4E*). The observed mTOR inhibitory effect of Sora-MNC would be beneficial for AML therapy because aberrations in mTOR pathway have been recognized to confer resistance to sorafenib and other FLT3 inhibitors [57, 58].

### Pharmacokinetics and biodistribution profiles

**Fig. 5:**
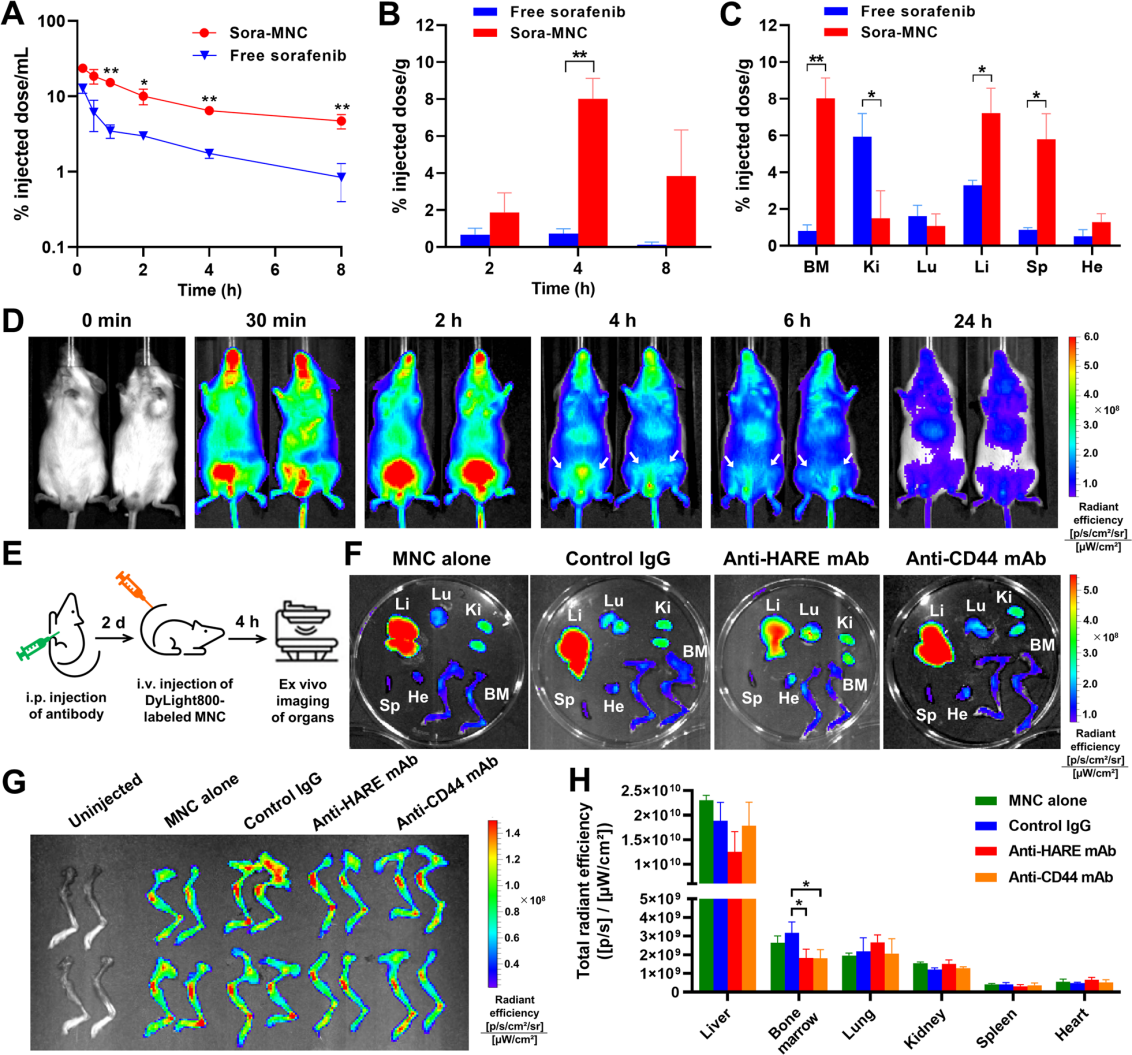
In vivo pharmacokinetics and biodistribution analysis. **A** Pharmacokinetics of Sora-MNC and free sorafenib in NSG mice. **B** Bone marrow accumulation of Sora-MNC as a function of time. **C** Biodistribution of Sora-MNC and free sorafenib at 4 h post-injection. *BM* bone marrow, *Ki* kidney, *Lu* lung, *Li* liver, *Sp* spleen, *He* heart. Mean ± SD (*n* = 3). **D** Time-dependent NIR fluorescence images of NSG mice following intravenous injection of DyLight800-labeled MNC. White arrows indicate the location of femur bones. **E** Experimental schedule for injection of blocking antibodies and DyLight800-labeled MNC. Ex vivo NIR fluorescence images of **F** organs and **G** femur and tibia bones harvested from the mice injected with different blocking antibodies and DyLight800-labeled MNC. **H** Quantification of the total radiant efficiency of the harvested organs. Mean ± SD (*n* = 4). ^*^P < 0.05; ^**^P < 0.005

We assessed pharmacokinetics and biodistribution of Sora-MNC in NOD-*scid Il2rg*^*-/-*^ (NSG) mice following intravenous administration. Sora-MNC exhibited significantly prolonged blood circulation compared to free sorafenib (Fig. 5A). Consistently, the area under the curve (AUC) of Sora-MNC was about 5-fold larger than that of sorafenib (Additional file 1: Table S3). Sora-MNC presented remarkably higher distribution in bone marrow than free sorafenib throughout the 8-h period, providing evidence of their bone marrow-homing capability (Fig. 5B). At 4 h post-injection, accumulation of Sora-MNC reached a peak of about 8% ID/g, which was about 11-fold greater than that of free sorafenib. This finding was in agreement with the previous study reporting the highest level of bone marrow deposition of ^3^ H-labelled HA (5.8% of ID/g) at 5 h post-injection [22], suggesting that HA plays an important role in the enhanced bone marrow targeting of Sora-MNC. Preferential accumulation of Sora-MNC was also observed in spleen and liver (Fig. 5C), which are the organs where infiltration of AML cells frequently occurs [59, 60]. In contrast, Sora-MNC showed lower deposition in kidney than free sorafenib, implying the potential of MNC to reduce sorafenib-induced nephrotoxicity.

The bone marrow-targeting ability of Sora-MNC was further examined by in vivo fluorescence imaging analysis. DyLight 800-labeled MNC emitting near-infrared (NIR) fluorescence was synthesized (Additional file 1: Fig. S8) and then intravenously administered into NSG mice via a tail vein. As shown in Fig. 5D, DyLight 800-labeled MNC showed diffuse distribution all over the body at early time points (30 min-2 h) and began to enrich in the femur bone area as early as 4 h post-injection. The whole-body fluorescence signal gradually decreased after 4 h, which was consistent with the observed pharmacokinetic profile. To understand the effect of HARE and CD44 on bone marrow accumulation of MNC, we pre-treated mice with either anti-HARE or anti-CD44 monoclonal antibody (mAb) or control IgG 2 days before intravenous administration of MNC (Fig. 5E). The ex vivo NIR imaging showed that pre-treatment of anti-HARE mAb caused a slight reduction in liver accumulation of MNC with a concomitant increase in its lung accumulation (Fig. 5F). On the other hand, anti-CD44 mAb and control IgG had little influence on the biodistribution of MNC. Interestingly, the accumulation of MNC in femur and tibia bones was decreased significantly (*P* < 0.05) upon pre-treatment of either anti-HARE or anti-CD44 mAb, as compared to control IgG group (Fig. 5G, H). Considering that bone marrow endothelial cells highly express HARE [18–20] and CD44 [61], the above results suggest that both HARE-HA and CD44-HA interactions play an important role in the enhanced accumulation of MNC in bone marrow niche.

### Therapeutic effect of Sora-MNC in vivo

**Fig. 6:**
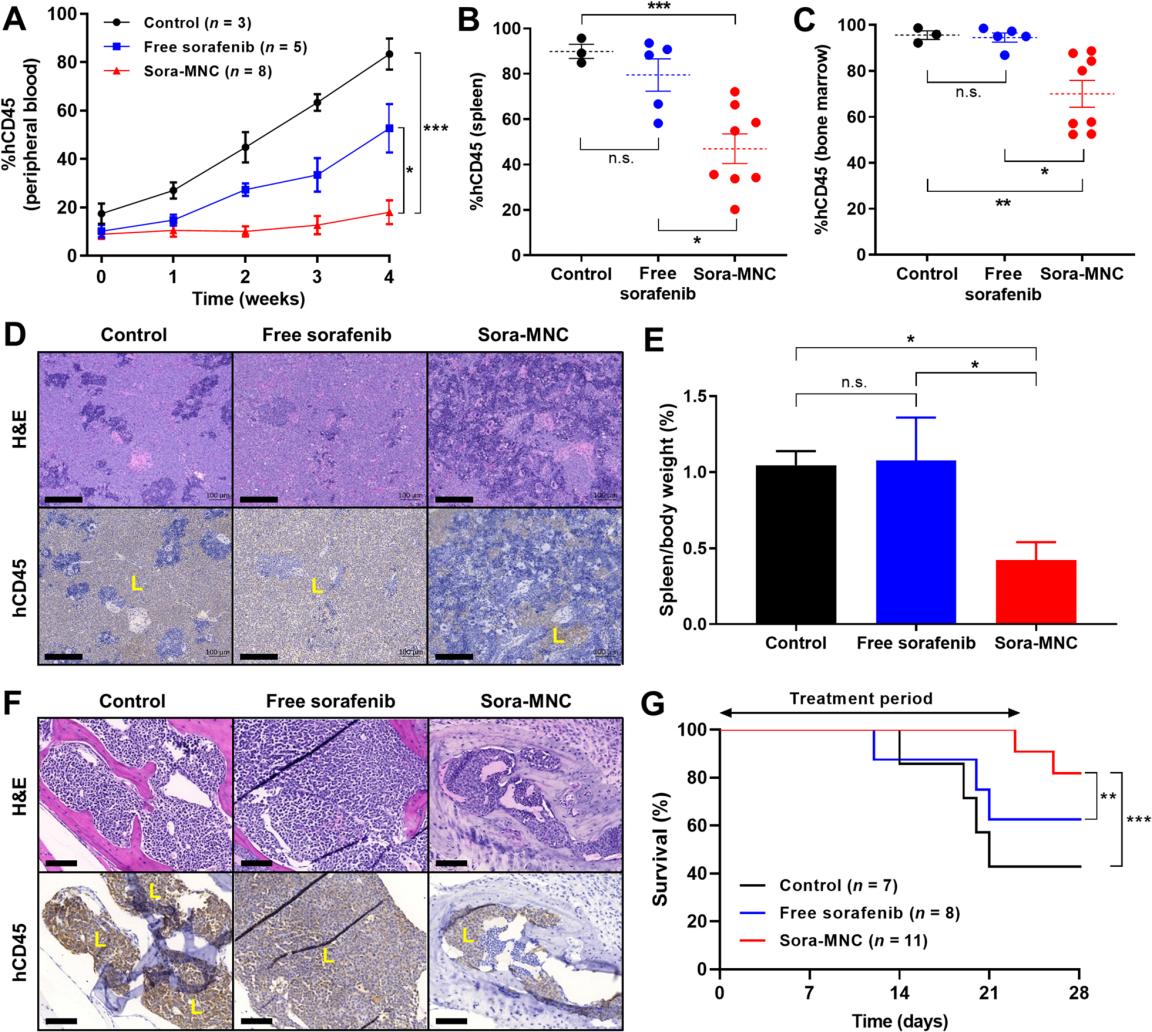
In vivo therapeutic efficacy study. **A** Time-course changes of the proportion of hCD45^+^ cells in the peripheral blood of AML-PDX mice subjected to different treatments. Proportion of hCD45^+^ cells in **B** spleen and **C** bone marrow at the end of treatments. **D** H&E and immunohistochemical staining of the spleen tissue harvested at the end of treatments. Human AML cells were identified with anti-hCD45 antibody (brown area labeled “L”). Scale bars, 200 μm. **E** Spleen-to-body weight ratios of different treatment groups at the 4-week endpoint. **F** High-magnification images of H&E and immunohistochemical staining of the bone marrow specimen. AML cells were identified with anti-hCD45 antibody (brown area labeled “L”). Scale bars, 100 μm. **G** Survival curves of AML-PDX mice subjected to different treatments; ^*^P < 0.05; ^**^P < 0.005; ^***^P < 0.0005; n.s.: nonsignificant

In vivo therapeutic efficacy of Sora-MNC was investigated in AML patient-derived xenograft (AML-PDX) mouse model [32]. NSG newborn pups were sub-lethally irradiated at 1 Gy and engrafted with patient-derived AML cells. When the proportion of hCD45^+^ AML cells in peripheral blood reached around 10–15%, the mice were randomly allocated for free sorafenib or Sora-MNC treatment at the same dose (0.4 mg kg^-1^) twice weekly for 4 weeks. Sora-MNC markedly suppressed the proliferation of hCD45^+^ AML cells in the peripheral blood, while AML progression was only slightly retarded by free sorafenib (Fig. 6A). At the 4-week endpoint, MNC-treated mice had much lower proportions of AML cells in the spleen and bone marrow than those treated with free sorafenib (Fig. 6B, C). The bone marrow of MNC-treated mice showed a less pronounced decrease in hCD45^+^ AML cells when compared to the spleen, presumably due to the increased drug resistance of leukemic blasts in the bone marrow niche through mechanisms involving cytokines and blast–stroma interactions [6, 7].

Our finding was further verified by histological assessment using H&E staining and immunohistochemistry (IHC). As shown in Fig. 6D, AML-PDX mice showed the abundant presence of hCD45^+^ cells (brown-stained area) in the spleen, indicating aggressive spread of leukemic blasts (Fig. 6D). Sora-MNC treatment caused a marked depletion of hCD45^+^ AML cells in the spleen, whereas no such effect was observed from free sorafenib. This finding was corroborated by a notable decrease in the spleen-to-body weight ratio, a well-known indicator of the leukemic burden in the spleen (Fig. 6E). Both control and free sorafenib-treated mice displayed significant build-up of AML cells in the bone marrow as indicated by strong nuclei stains (blue/purple) by H&E as well as positive stains for hCD45 by IHC (Fig. 6F and Additional file 1: S11). On the other hand, bone marrow sections of mice treated with Sora-MNC displayed regions of AML cells undergoing cell death, as depicted by decolorization of the nucleus (H&E) with very poor and scattered hCD45^+^ (IHC) stained cells. These results revealed that Sora-MNC diminished the leukemic burden in the bone marrow more effectively than free sorafenib.

A significantly improved survival was also observed in MNC-treated mice compared to those treated with free sorafenib (Fig. 6G). All the mice treated with Sora-MNC survived during the treatment period for 23 days, whereas those treated with free sorafenib started to die earlier on day 12. Blood chemistry analysis revealed that free sorafenib caused noticeable liver and kidney damage, as evident from the decreased albumin:globulin ratio and increased blood urea nitrogen levels, respectively (Additional file 1: Fig. S12). In contrast, the mice treated with Sora-MNC had similar albumin:globulin ratio and blood urea nitrogen levels to those of untreated mice, reflecting its negligible hepatotoxicity and nephrotoxicity. There was no significant change in serum potassium and sodium levels in the mice treated with Sora-MNC compared to control group (Additional file 1: Fig. S13), showing no sign of electrolyte disturbance and cardiac toxicity [62]. Collectively, these results demonstrated superior therapeutic efficacy and safety of Sora-MNC over free sorafenib formulation in AML-PDX model.

## Conclusion

Our study presented, for the first time, the development of a bone marrow-targetable sorafenib nanomedicine for AML therapy. Sorafenib-loaded micellar nanocomplex (Sora-MNC) was formed by self-assembly of sorafenib and HA-EGCG in aqueous solution, and subsequently purified by centrifugal filtration technique. Sora-MNC formulations were much more effective in killing FLT3-mutated AML cells than free sorafenib at equivalent doses. RNA-seq and qPCR analysis revealed that Sora-MNC synergistically enhanced the anti-leukemic activity of sorafenib through effective inhibition of mTOR signaling pathway and its downstream molecules. Moreover, pharmacokinetics and biodistribution analysis demonstrated prolonged systemic circulation and improved bone marrow accumulation of Sora-MNC upon intravenous administration. In AML-PDX mouse model, Sora-MNC eradicated bone marrow-residing leukemic blasts and extended the period of survival more effectively than free sorafenib without harmful side effects. The current study may provide useful information that promotes the development of nanomedicine platforms targeting diverse therapeutic agents into bone marrow niche for more effective treatment of AML and other bone marrow disorders.

## Supplementary Information

**Additional file 1:** **Figure S1.** Representative TEM images of (A) Sora-MNC-2 and (B) Sora-MNC-3. **Figure S2.** Zeta potential distribution profiles of (A) Sora-MNC formulations and (B) HA-EGCG, HA and sorafenib. HA-EGCG (10 mg mL^−1^) and HA (10 mg mL^−1^) were dissolved in deionized water. Sorafenib was first dissolved in methanol at 1 mg mL^−1^ and then 10-fold diluted with water for the zeta potential measurement. **Figure S3.** Stability of HA-EGCG micelles in 10% (v/v) fetal bovine serum over 5 days. Mean ± SD (n = 3). **Figure S4.** Anti-leukemic activity of Sora-MNC-1, HA-EGCG and EGCG on (A) MOLM-14 and (B) MV-4-11 cells as a function of EGCG unit concentration. Mean ± SD (n = 4). **Figure S5.** Median effect plots showing eradication of (A) MOLM-14 and (B) MV-4-11 cells treated with free sorafenib, HA-EGCG or their combination (Sora-MNC-1). Cytotoxicity data were plotted using the linearized median effect equation, log(fa/fu) = m log(D) – m log (Dm), where fa is the fraction of killed cells, fu is the fraction of survived cells, D is the dose applied, Dm is the median effective dose. The resultant plot gave the slope of m and the y-axis intercept of – m log (Dm). **Figure S6.** Hemolysis assay. (A) Representative photograph of mouse red blood cells treated with PBS, Triton X-100 (1%), or Sora-MNC-1 at various sorafenib doses for 1 h at 37 °C. (B) Effect of different treatments on the absorbance at 576 nm (an indicator of hemoglobin leakage from red blood cells). **Figure S7.** Flow cytometric detection of CD44 in MOLM-14, MV-4-11 and bone marrow stromal cells labeled without (left panel) or with FITC-tagged anti-CD44 antibody (right panel). **Figure S8.** (A) Z-average sizes and zeta potential values of Sora-MNC, DyLight 488-labeled MNC and DyLight 800-labeled MNC. (B) Fluorescence emission spectra (λex=400 nm) of DyLight 488 dye (0.312 µg mL^−1^) and DyLight 488-labeled MNC (Sorafenib concentration=0.8 µg mL^−1^). (C) Fluorescence emission spectra (λex=750 nm) of DyLight 800 dye (2.5 µg mL^−1^) and DyLight 800-labeled MNC (Sorafenib concentration=4 µg mL^−1^). **Figure S9.** KEGG map of differentially expressed genes (DEGs) involved in the tricarboxylic acid (TCA) cycle. The gradient color scale indicates upregulation (red) and downregulation (green) of gene expression calculated as log2 fold change. The names of DEGs selected for qPCR analysis are highlighted in blue. FH: Fumarate hydratase, SDHA: succinate dehydrogenase, OGDH: 2-oxoglutarate dehydrogenase, IDH1: isocitrate dehydrogenase 1. **Figure S10.** KEGG map of DEGs involved in the aminoacyl-tRNA biosynthesis. The gradient color scale indicates upregulation (red) and downregulation (green) of gene expression calculated as log2 fold change. The names of DEGs selected for qPCR analysis are highlighted in blue. LARS: leucyl-tRNA synthetase, FARSA: phenylalanyl-tRNA synthetase. **Figure S11.** Low-magnification images of H&E and immunohistochemical staining of the bone marrow specimen. AML cells were identified with anti-hCD45 antibody (brown area labeled “L”). Scale bars, 200 μm. The red boxes indicate the areas of the higher magnification images included in Figure 6F.** Figure S12.** (A) Albumin:globulin ratio and (B) blood urea nitrogen level in the experimental groups. Mean ± SD (n = 3); *P < 0.05; n.s.=nonsignificant.** Figure S13.** (A) Potassium and (B) sodium ion level in the experimental groups. Mean ± SD (n = 3); n.s.=nonsignificant. **Table S1.** Characteristics of Sora-MNC selected for in vitro studies. **Table S2.** Sequence of the primers used for qPCR analysis. **Table S3.** Pharmacokinetic parameters of Sora-MNC and free sorafenib.

## Data Availability

The datasets analysed during the current study are available from the corresponding author on reasonable request.
