# Supplementary material for: Bone marrow-targetable Green Tea Catechin-Based Micellar Nanocomplex for synergistic therapy of Acute myeloid leukemia
Source: J Nanobiotechnology. 2022 Nov 16;20:481. doi: 10.1186/s12951-022-01683-4 (PMC9670631; doi:10.1186/s12951-022-01683-4)
Supplement: Supplementary file 1 — Additional file 1: Figure S1. Representative TEM images of (A) Sora-MNC-2 and (B) Sora-MNC-3. Figure S2. Zeta potential distribution profiles of (A) Sora-MNC formulations and (B) HA-EGCG, HA and sorafenib. HA-EGCG (10 mg mL−1) and HA (10 mg mL−1) were dissolved in deionized water. Sorafenib was first dissolved in methanol at 1 mg mL−1 and then 10-fold diluted with water for the zeta potential measurement. Figure S3. Stability of HA-EGCG micelles in 10% (v/v) fetal bovine serum over 5 days. Mean ± SD (n = 3). Figure S4. Anti-leukemic activity of Sora-MNC-1, HA-EGCG and EGCG on (A) MOLM-14 and (B) MV-4-11 cells as a function of EGCG unit concentration. Mean ± SD (n = 4). Figure S5. Median effect plots showing eradication of (A) MOLM-14 and (B) MV-4-11 cells treated with free sorafenib, HA-EGCG or their combination (Sora-MNC-1). Cytotoxicity data were plotted using the linearized median effect equation, log(fa/fu) = m log(D) – m log (Dm), where fa is the fraction of killed cells, fu is the fraction of survived cells, D is the dose applied, Dm is the median effective dose. The resultant plot gave the slope of m and the y-axis intercept of – m log (Dm). Figure S6. Hemolysis assay. (A) Representative photograph of mouse red blood cells treated with PBS, Triton X-100 (1%), or Sora-MNC-1 at various sorafenib doses for 1 h at 37 °C. (B) Effect of different treatments on the absorbance at 576 nm (an indicator of hemoglobin leakage from red blood cells). Figure S7. Flow cytometric detection of CD44 in MOLM-14, MV-4-11 and bone marrow stromal cells labeled without (left panel) or with FITC-tagged anti-CD44 antibody (right panel). Figure S8. (A) Z-average sizes and zeta potential values of Sora-MNC, DyLight 488-labeled MNC and DyLight 800-labeled MNC. (B) Fluorescence emission spectra (λex=400 nm) of DyLight 488 dye (0.312 µg mL−1) and DyLight 488-labeled MNC (Sorafenib concentration=0.8 µg mL−1). (C) Fluorescence emission spectra (λex=750 nm) of DyLight 800 dye (2.5 µg mL−1) an [file 12951_2022_1683_MOESM1_ESM.doc]

***Supporting Information for***

Bone Marrow-Targetable Green Tea Catechin-Based Micellar Nanocomplex for Synergistic Therapy of Acute Myeloid Leukemia

Ki Hyun Bae^1†^, Fritz Lai^2†^, Jamie Mong^1^, Akiko Nambu^3^, Kiat Hwa Chan^4^, Zhisheng Her^2^, Motomi Osato^3^, Min-Han Tan^1^, Qingfeng Chen^2*^ and Motoichi Kurisawa^1*^

^1^Institute of Bioengineering and Bioimaging, 31 Biopolis Way, The Nanos, Singapore 138669.

^2^Institute of Molecular and Cell Biology, 61 Biopolis Drive, The Proteos, Singapore 138673.

^3^Cancer Science Institute of Singapore, National University of Singapore, 14 Medical Drive, Singapore 117599.

^4^Division of Science, Yale-NUS College, 16 College Avenue West, Singapore 138527.

*Corresponding author. Email: kurisawa@jaist.ac.jp (M.K.); qchen@imcb.a-star.edu.sg (Q.C.)


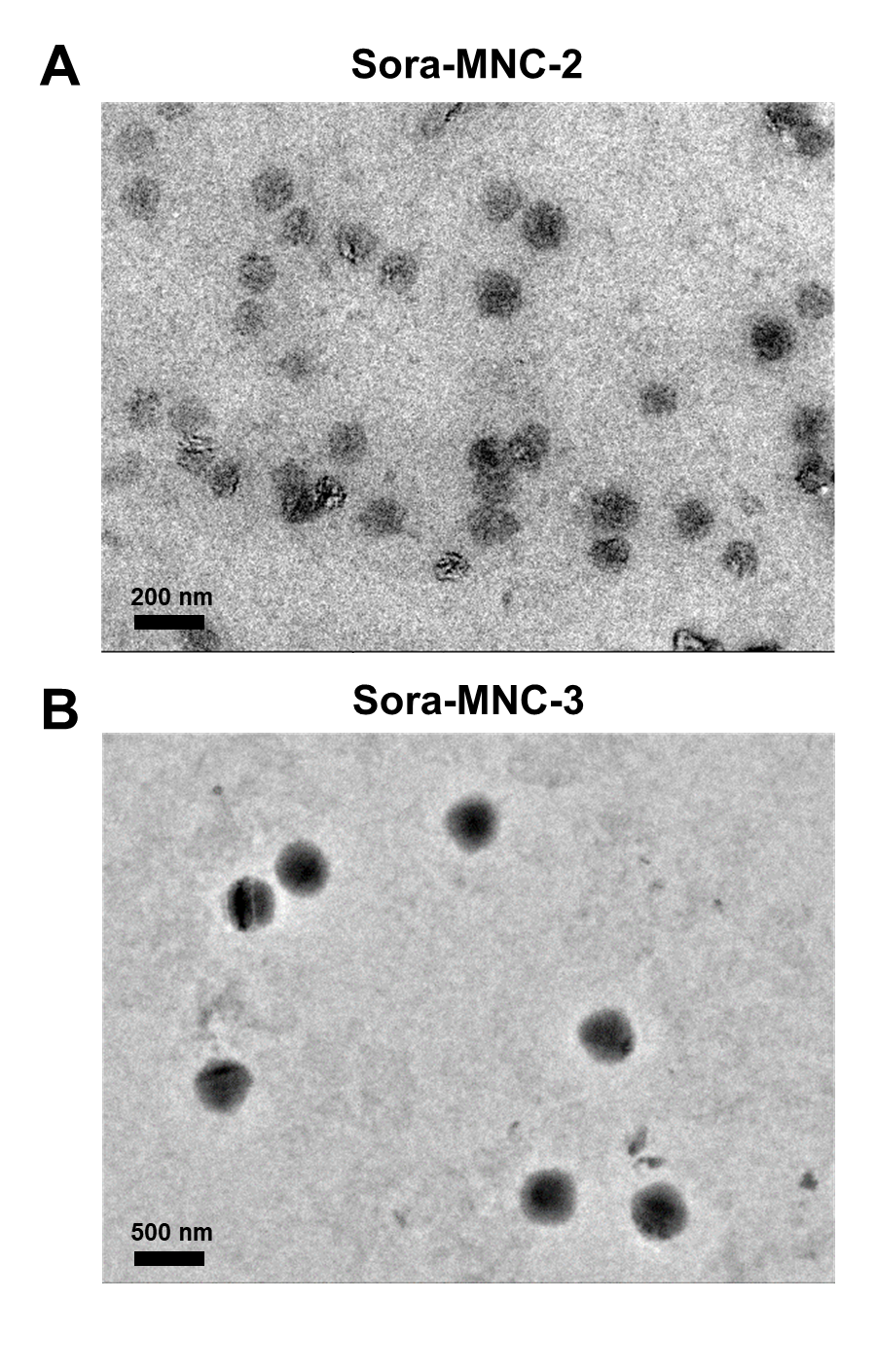


**Figure S1**. Representative TEM images of (A) Sora-MNC-2 and (B) Sora-MNC-3.


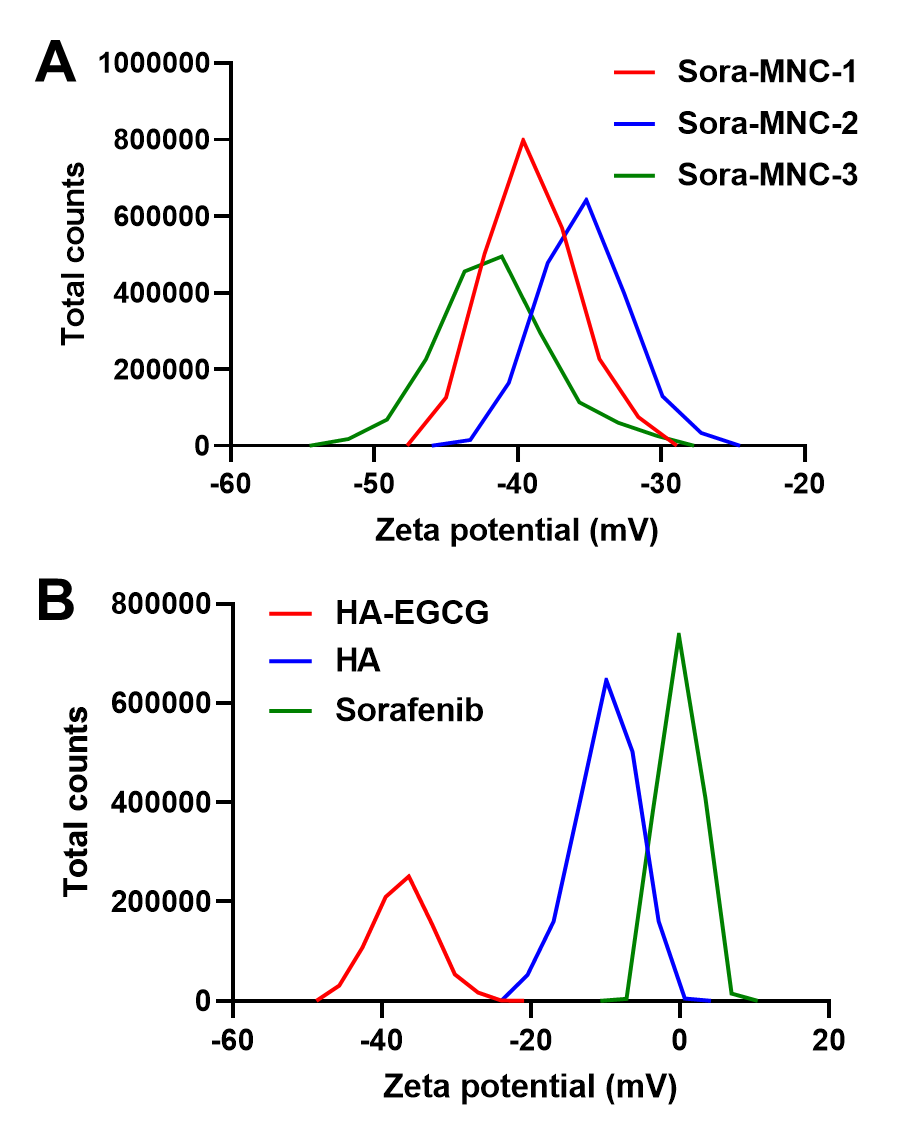


**Figure S2**. Zeta potential distribution profiles of (A) Sora-MNC formulations and (B) HA-EGCG, HA and sorafenib. HA-EGCG (10 mg mL^-1^) and HA (10 mg mL^-1^) were dissolved in deionized water. Sorafenib was first dissolved in methanol at 1 mg mL^-1^ and then 10-fold diluted with water for the zeta potential measurement.


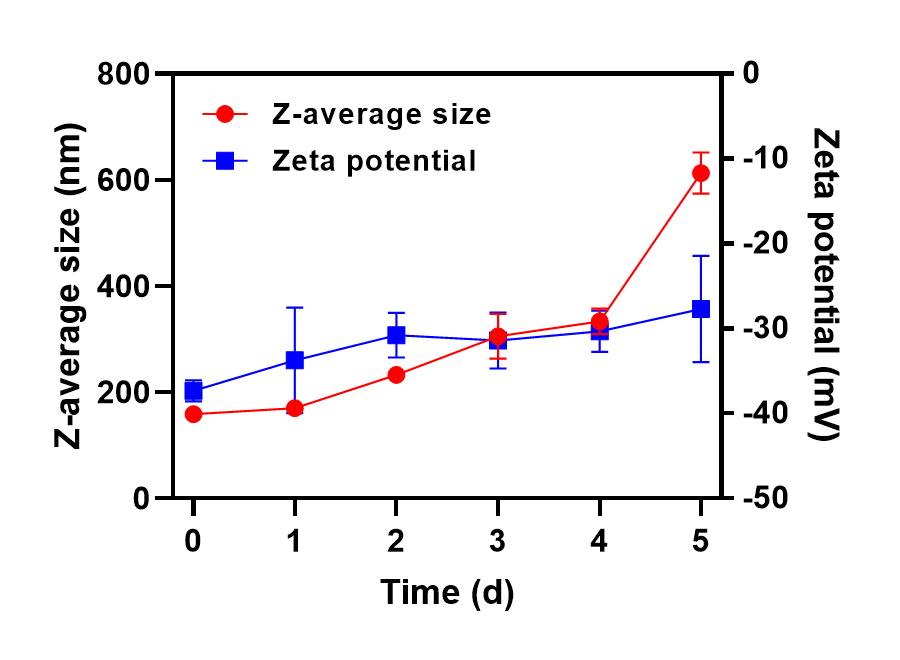


**Figure S3**. Stability of HA-EGCG micelles in 10% (v/v) fetal bovine serum over 5 days. Mean ± SD (*n* = 3).


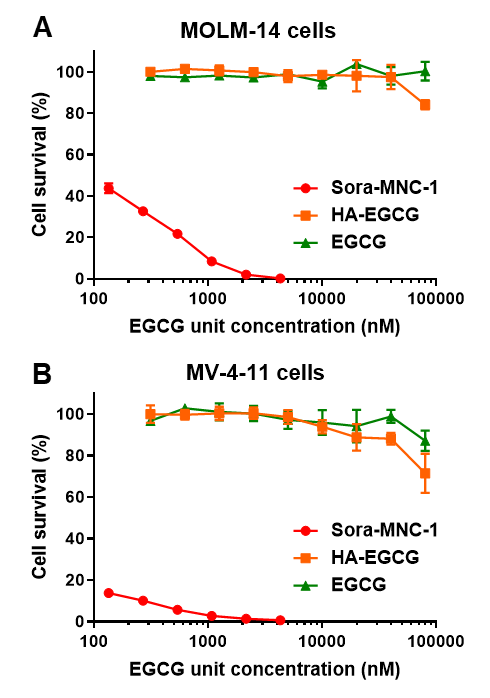


Figure S4. Anti-leukemic activity of Sora-MNC-1, HA-EGCG and EGCG on (A) MOLM-14 and (B) MV-4-11 cells as a function of EGCG unit concentration. Mean ± SD (*n* = 4).


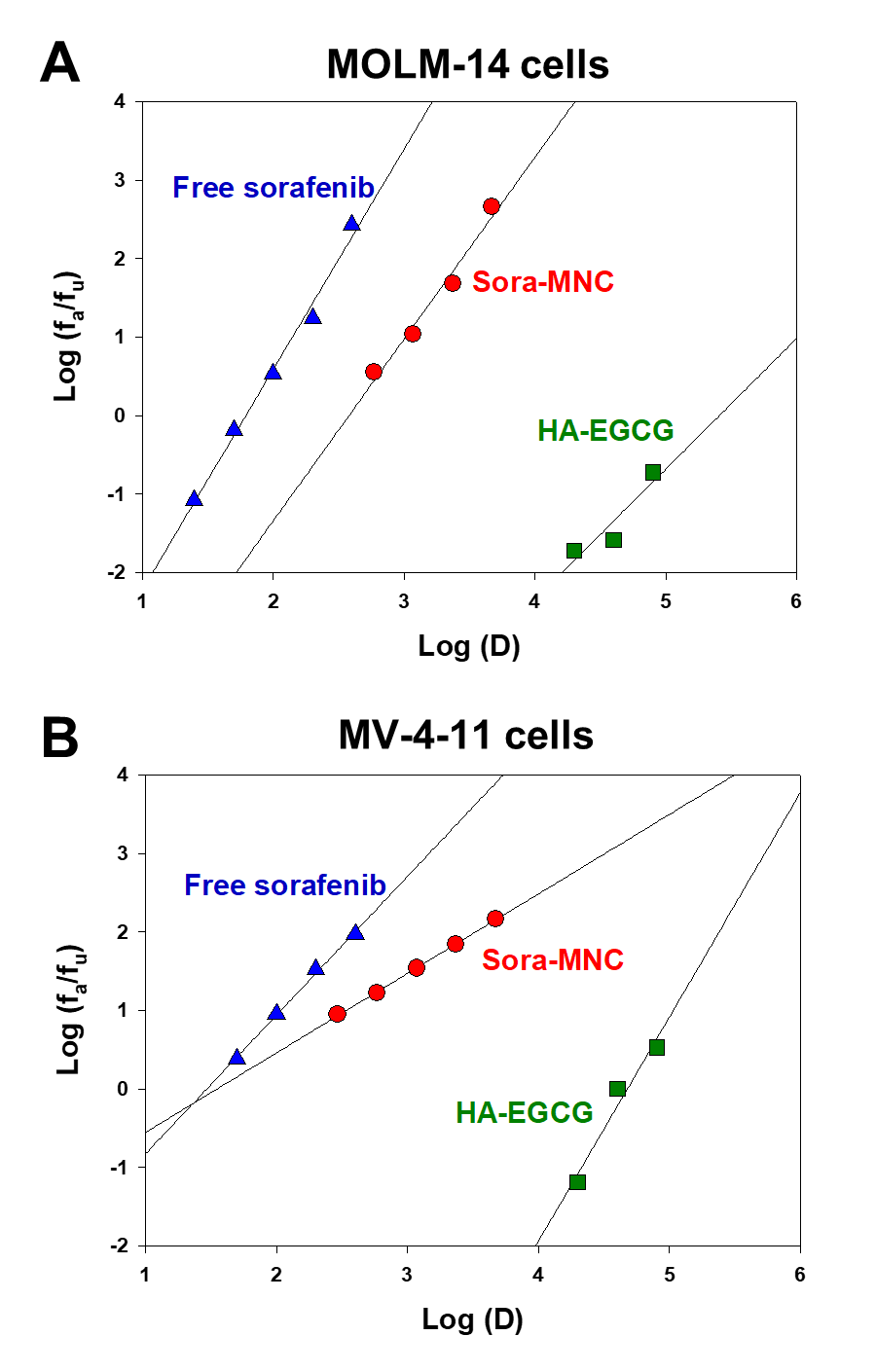


**Figure S5.** Median effect plots showing eradication of (A) MOLM-14 and (B) MV-4-11 cells treated with free sorafenib, HA-EGCG or their combination (Sora-MNC-1). Cytotoxicity data were plotted using the linearized median effect equation, log(f_a_/f_u_) = m log(D) – m log (D_m_), where f_a_ is the fraction of killed cells, f_u_ is the fraction of survived cells, D is the dose applied, D_m_ is the median effective dose. The resultant plot gave the slope of m and the y-axis intercept of – m log (D_m_).


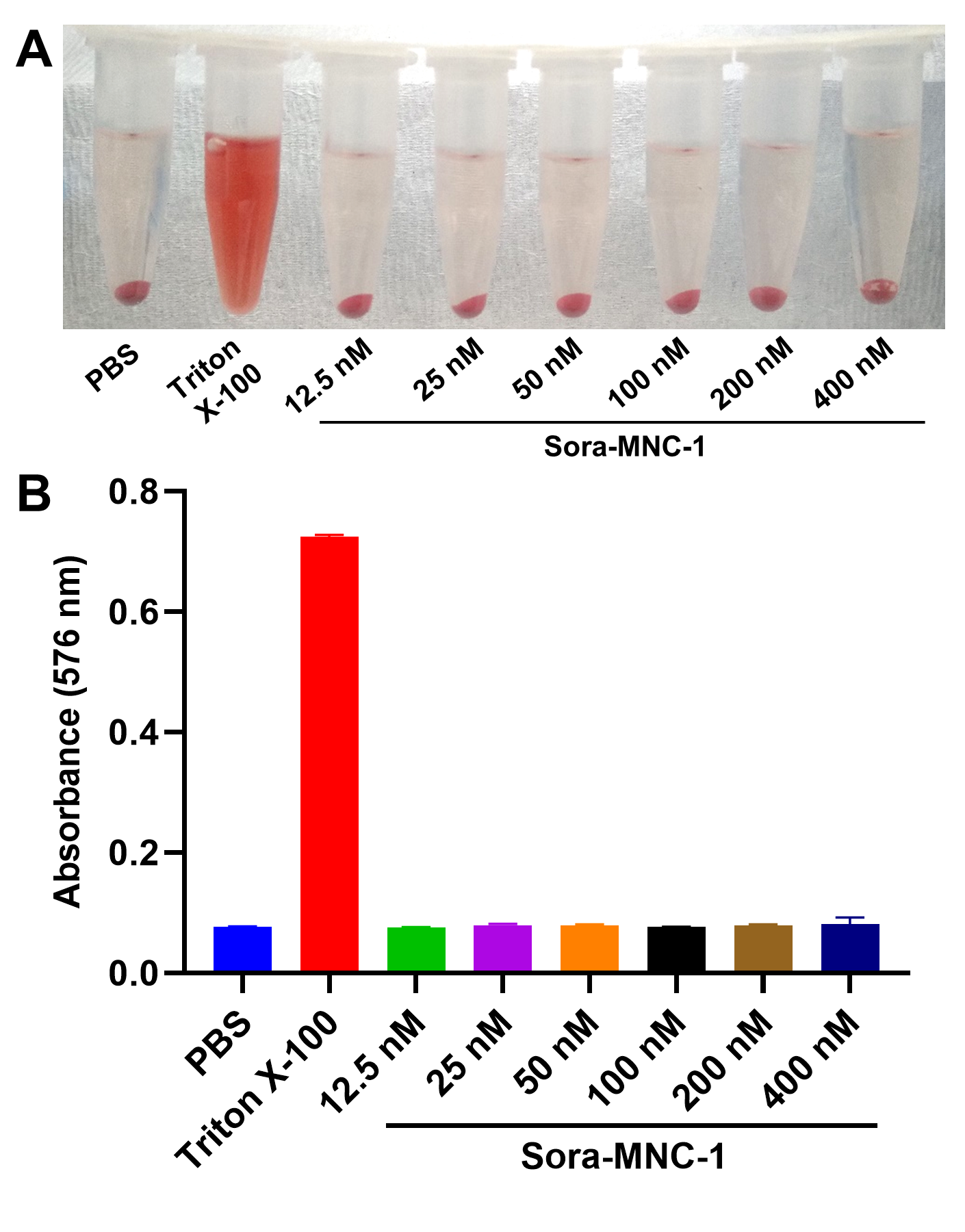


**Figure S6.** Hemolysis assay. (A) Representative photograph of mouse red blood cells treated with PBS, Triton X-100 (1%), or Sora-MNC-1 at various sorafenib doses for 1 h at 37 °C. (B) Effect of different treatments on the absorbance at 576 nm (an indicator of hemoglobin leakage from red blood cells).


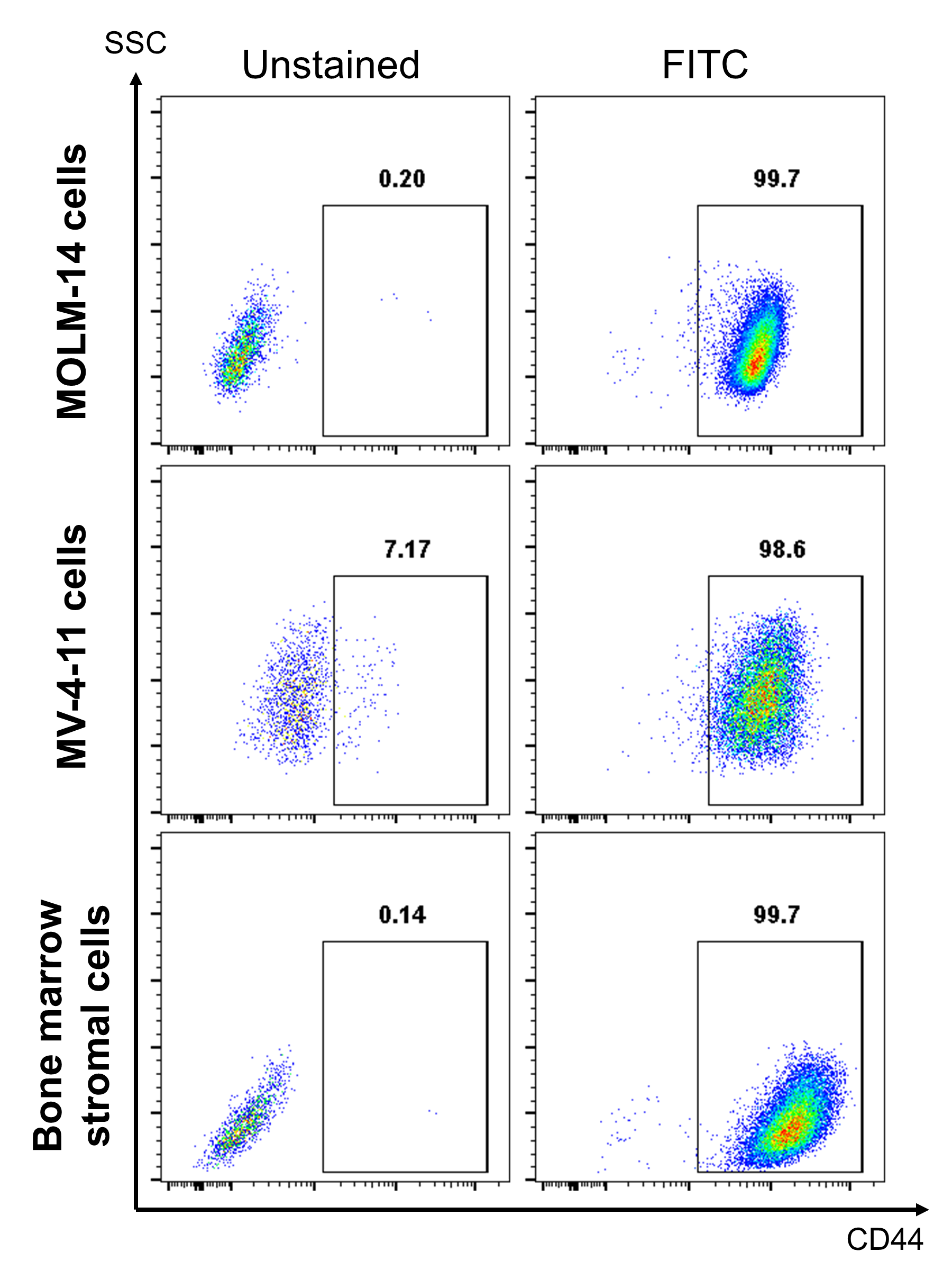


**Figure S7.** Flow cytometric detection of CD44 in MOLM-14, MV-4-11 and bone marrow stromal cells labeled without (left panel) or with FITC-tagged anti-CD44 antibody (right panel).


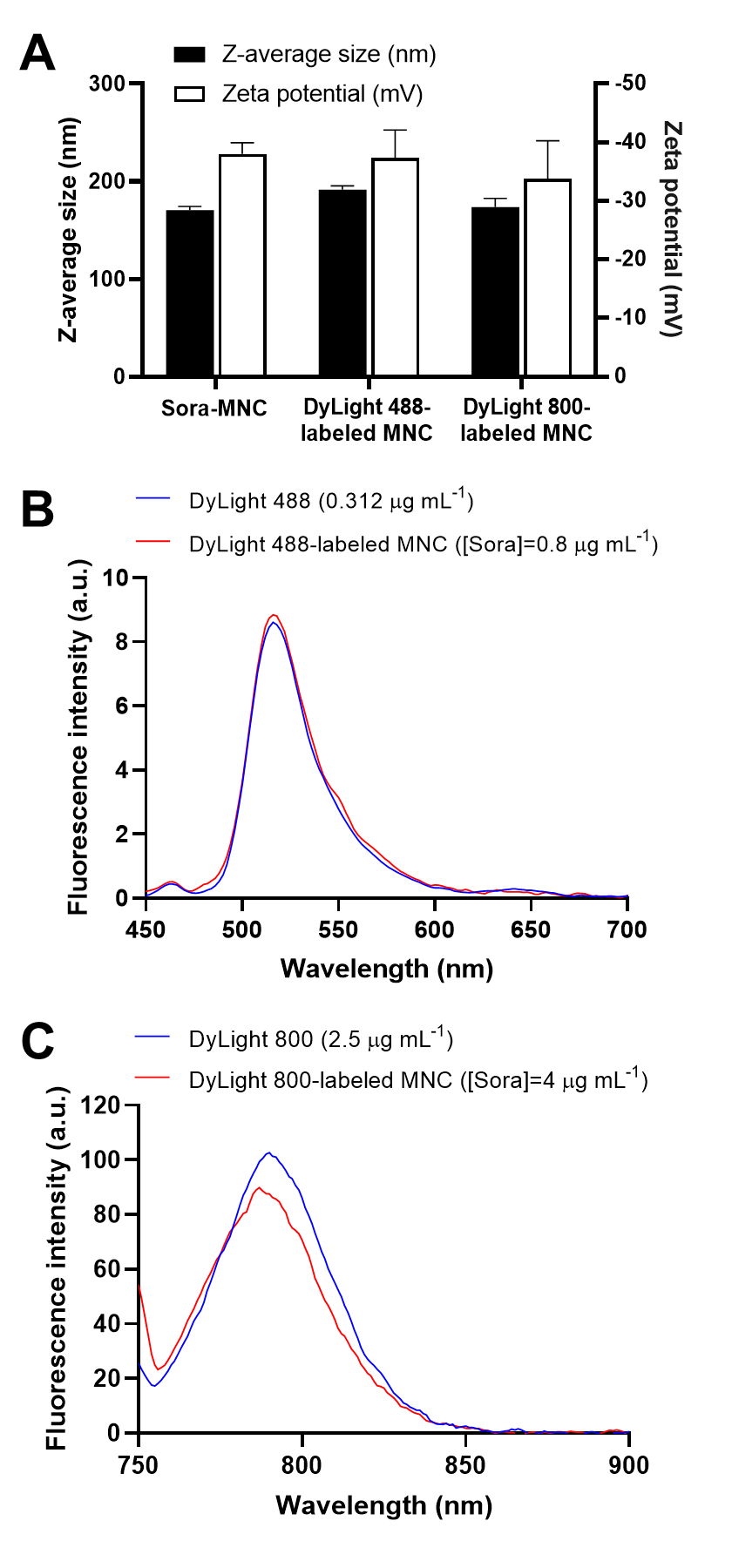


**Figure S8.** (A) Z-average sizes and zeta potential values of Sora-MNC, DyLight 488-labeled MNC and DyLight 800-labeled MNC. (B) Fluorescence emission spectra (λ_ex_=400 nm) of DyLight 488 dye (0.312 µg mL^-1^) and DyLight 488-labeled MNC (Sorafenib concentration=0.8 µg mL^-1^). (C) Fluorescence emission spectra (λ_ex_=750 nm) of DyLight 800 dye (2.5 µg mL^-1^) and DyLight 800-labeled MNC (Sorafenib concentration=4 µg mL^-1^).


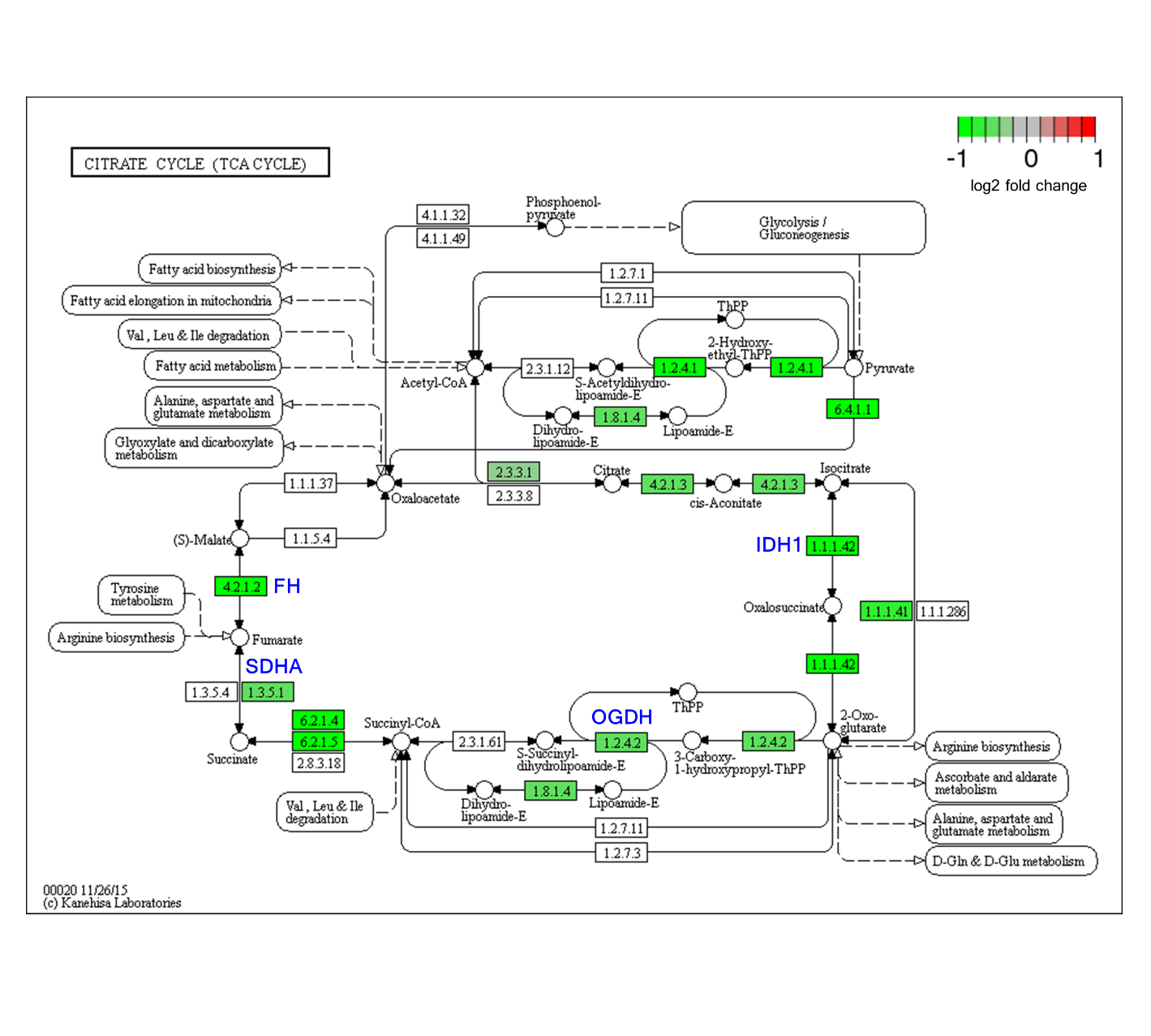


**Figure S9.** KEGG map of differentially expressed genes (DEGs) involved in the tricarboxylic acid (TCA) cycle. The gradient color scale indicates upregulation (red) and downregulation (green) of gene expression calculated as log2 fold change. The names of DEGs selected for qPCR analysis are highlighted in blue. FH: Fumarate hydratase, SDHA: succinate dehydrogenase, OGDH: 2-oxoglutarate dehydrogenase, IDH1: isocitrate dehydrogenase 1.

**
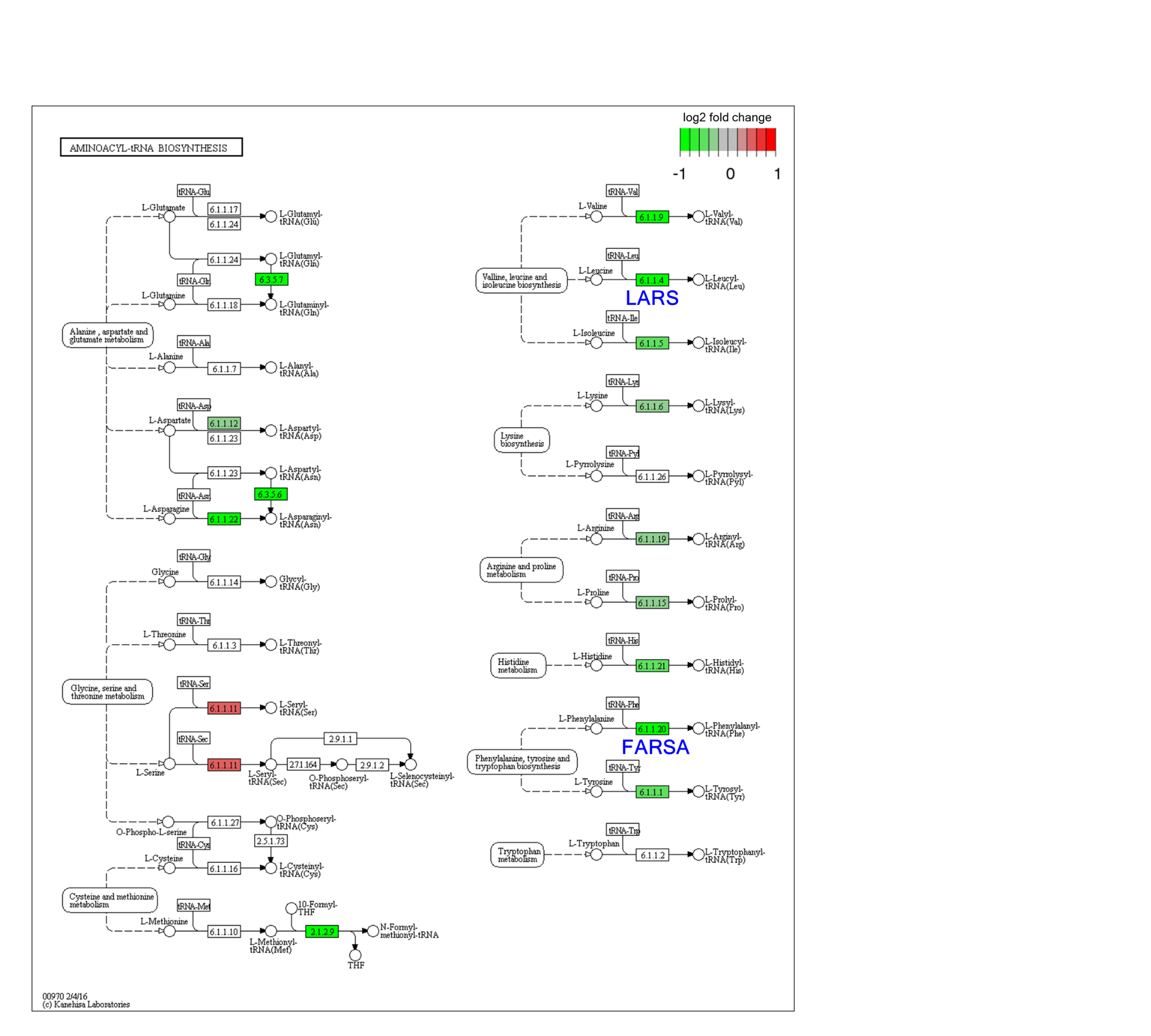
**

**Figure S10.** KEGG map of DEGs involved in the aminoacyl-tRNA biosynthesis. The gradient color scale indicates upregulation (red) and downregulation (green) of gene expression calculated as log2 fold change. The names of DEGs selected for qPCR analysis are highlighted in blue. LARS: leucyl-tRNA synthetase, FARSA: phenylalanyl-tRNA synthetase.


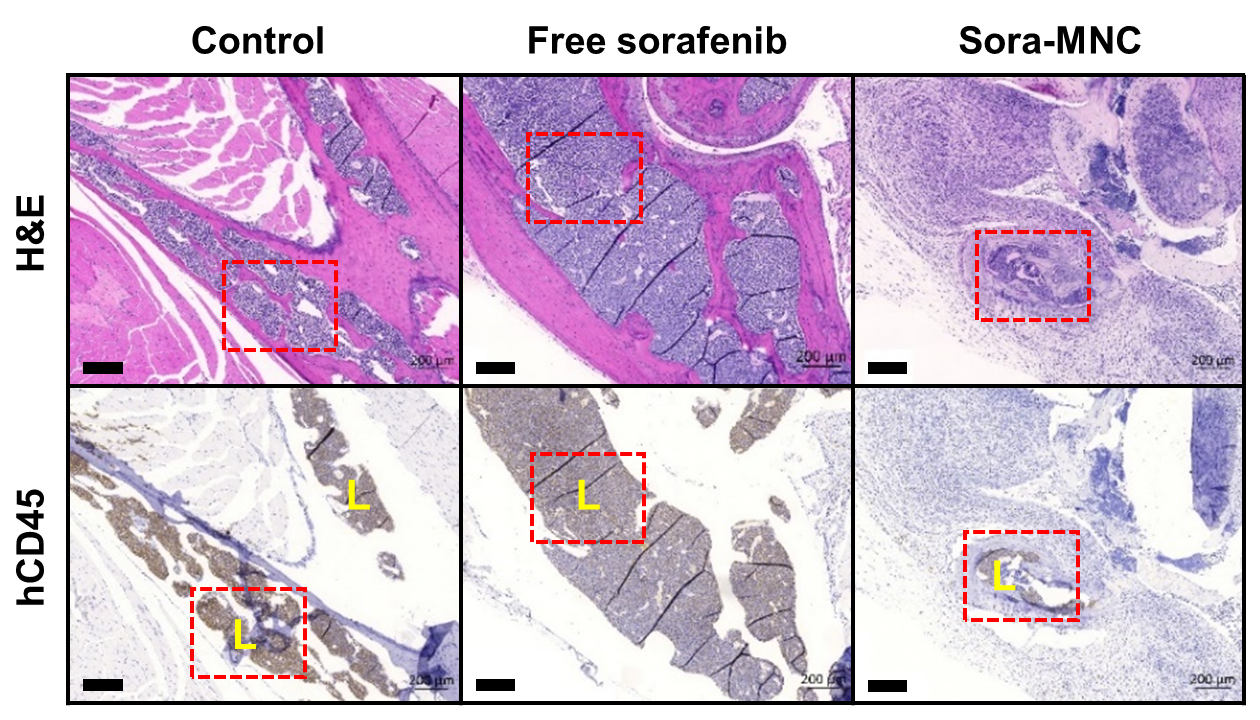


**Figure S11.** Low-magnification images of H&E and immunohistochemical staining of the bone marrow specimen. AML cells were identified with anti-hCD45 antibody (brown area labeled “L”). Scale bars, 200 μm. The red boxes indicate the areas of the higher magnification images included in Figure 6F.

**
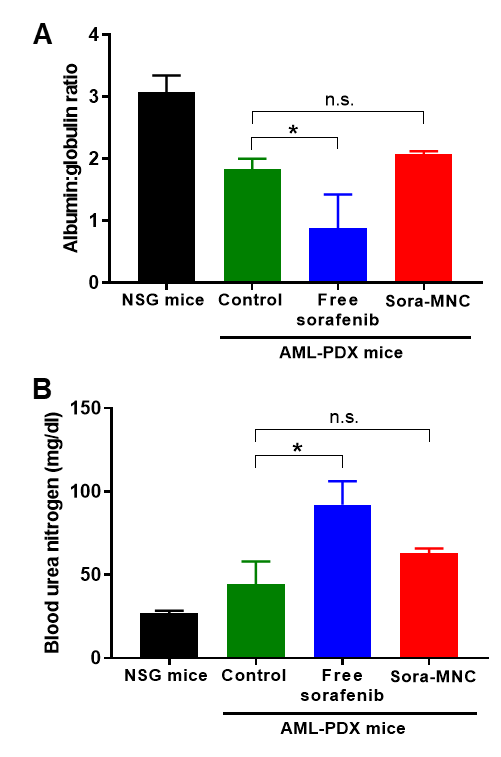
**

**Figure S12.** (A) Albumin:globulin ratio and (B) blood urea nitrogen level in the experimental groups. Mean ± SD (*n* = 3); ^*^*P* < 0.05; n.s.=nonsignificant.

**
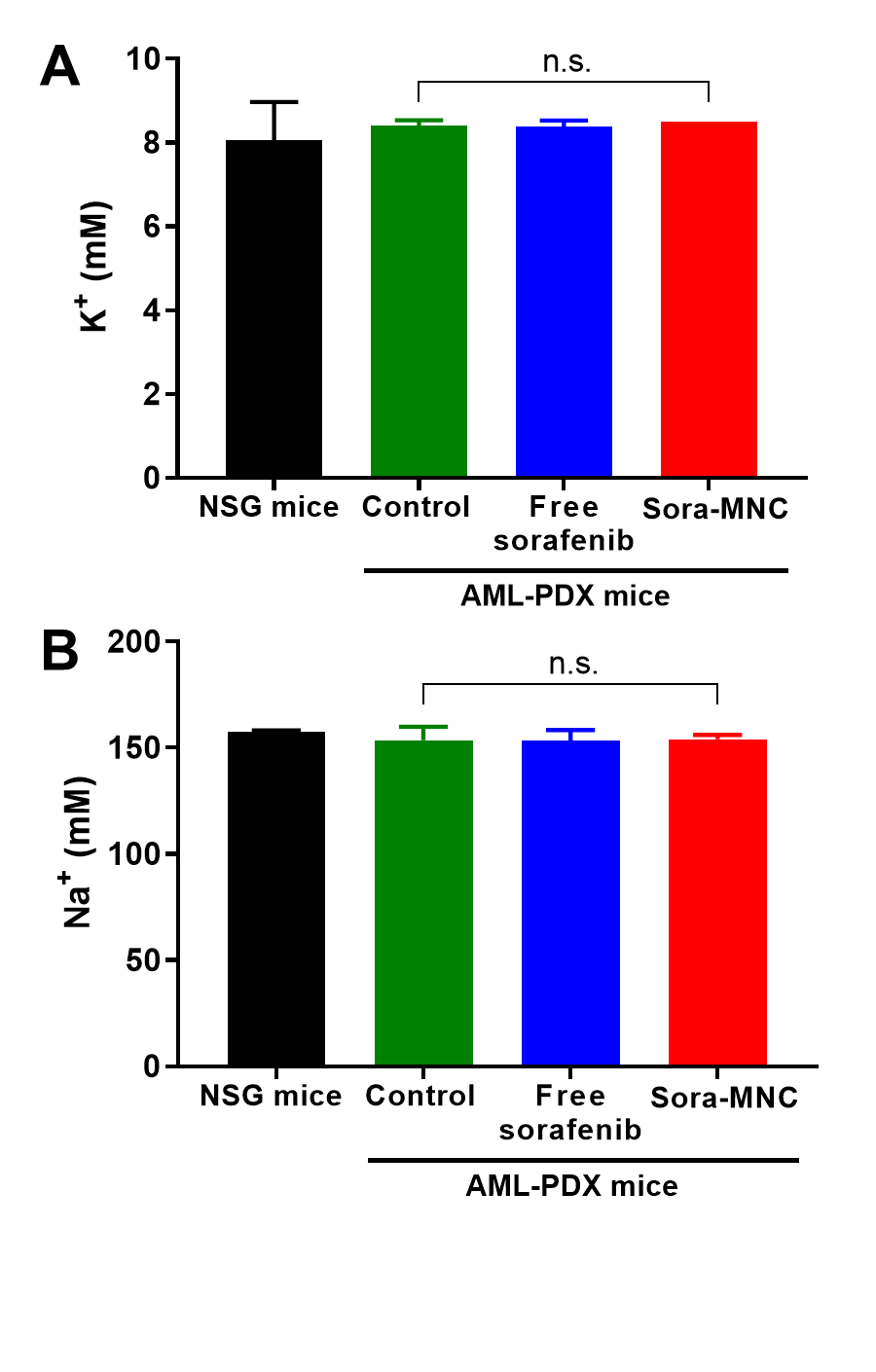
**

**Figure S13.** (A) Potassium and (B) sodium ion level in the experimental groups. Mean ± SD (*n* = 3); n.s.=nonsignificant.

Table S1.

Characteristics of Sora-MNC selected for *in vitro* studies.

| Sample code | Initial HA-EGCG concentration  (mg mL^-1^) | Initial sorafenib concentration  (mg mL^-1^) | Peak diameter (nm) | Zeta potential (mV) | Drug loading efficiency (%) | Drug loading content (wt%) |
| --- | --- | --- | --- | --- | --- | --- |
| Sora-MNC-1 | 8 | 0.05 | 122 ± 3.63 | -38.6 ± 1.94 | 21.4 ± 1.02 | 0.32 ± 0.03 |
| Sora-MNC-2 | 8 | 0.1 | 190 ± 4.22 | -37.3 ± 1.12 | 52.8 ± 0.05 | 1.52 ± 0.05 |
| Sora-MNC-3 | 4 | 0.1 | 342 ± 3.39 | -42.8 ± 1.58 | 49.3 ± 3.13 | 4.27 ± 0.10 |

Table S2.

Sequence of the primers used for qPCR analysis.

| Gene | Forward (5' to 3') | Reverse (5' to 3') | Product Size | NCBI Ref Seq |
| --- | --- | --- | --- | --- |
| *FH* | GCACCATGTACCGAGCACTT | GGAATTTTGGCTTGCCATTCG | 152 | NM_000143.4 |
| *OGDH* | AGGGCATATCAGGTCAGGGG | GCCATAGAACCCCACGTTTGA | 114 | NM_001165036.2 |
| *SDHA* | CTCAGCATGCAGAAGGTGCG | AAGTTTTGTCGATCACGGGTCT | 166 | NM_001330758.2 |
| *IDH1* | TGCCTGGAGTTTAAAAGGCGA | CTCTACCACAGAACCGCCAC | 143 | NM_001282386.1 |
| *FARSA* | AGAGCGAGCTTATGCGACTG | TGTCCACCCGAATCCACTTG | 78 | NM_004461.3 |
| *LARS* | GTCGTGGATTTGCACAGTCG | TACTTGCCCTTGCTGGTCTG | 170 | NM_020117.11 |
| *EIF4E* | GACTGTCGAACCGGGTG | CAGGGTAATCATGGAGGAGGC | 86 | NM_001331017.2 |
| *RPS6KB1* | AGGGGGCTATGGAAAGGCAA | TCCACCAGTCTGAAAGGCAT | 133 | NM_001272042.2 |
| *ACTB* | CTGGCACCACACCTTCTACA | TAGCACAGCCTGGATAGCAA | 166 | NM_001101.3 |

Table S3.

Pharmacokinetic parameters of Sora-MNC and free sorafenib.

| Parameter* | Free sorafenib | Sora-MNC |
| --- | --- | --- |
| *t*_1/2α_ (h) | 0.16 ± 0.11 | 0.52 ± 0.29 |
| *t*_1/2β_ (h) | 4.05 ± 1.18 | 8.98 ± 5.37 |
| MRT (h) | 4.74 ± 1.44 | 11.2 ± 7.00 |
| *V*_1_ (mL) | 3.67 ± 1.58 | 2.73 ± 1.50 |
| *V*_2_ (mL) | 14.0 ± 3.59 | 5.04 ± 2.06 |
| *V*_ss_ (mL) | 17.7 ± 4.76 | 7.78 ± 3.09 |
| *Cl*_1_ (mL h^-1^) | 3.77 ± 0.24 | 0.77 ± 0.15 |
| *Cl*_2_ (mL h^-1^) | 11.9 ± 4.94 | 2.10 ± 0.42 |
| AUC_0-∞_ (µg·h mL^-1^) | 2.66 ± 0.17 | 13.6 ± 3.20 |

*The distribution half-life (*t*_1/2α_), elimination half-life (*t*_1/2β_), mean residence time (MRT), central volume of distribution (*V*_1_), peripheral volume of distribution (*V*_2_), apparent volume of distribution at steady-state (*V*_ss_), central clearance (*Cl*_1_), peripheral clearance (*Cl*_2_), and area under the curve (AUC_0-∞_) were calculated by using a two-compartment model with the PKSolver software. Mean ± SD (*n* = 4).
